# Host Species and Environment Shape the Gut Microbiota of Cohabiting Marine Bivalves

**DOI:** 10.1007/s00248-023-02192-z

**Published:** 2023-02-22

**Authors:** Shirin Akter, Melissa L Wos-Oxley, Sarah R Catalano, Md Mahbubul Hassan, Xiaoxu Li, Jian G Qin, Andrew PA Oxley

**Affiliations:** 1 College of Science and Engineering, Flinders University, Adelaide, SA Australia; 2 School of Medicine, Deakin University, Geelong, VIC Australia; 3 Aquatic Sciences Centre, South Australian Research and Development Institute, West Beach, SA Australia; 4 Aquaculture Research and Development, Department of Primary Industries and Regional Development, Hillarys, WA Australia; 5 School of Life and Environmental Sciences, Deakin University, Geelong, VIC Australia

**Keywords:** Gut microbiota, Seasonal changes, Oysters, Mussels, Aquaculture, *Mycoplasma*

## Abstract

Pacific oysters (*Crassostrea gigas*) and Mediterranean mussels (*Mytilus galloprovincialis*) are commercially important marine bivalves that frequently coexist and have overlapping feeding ecologies. Like other invertebrates, their gut microbiota is thought to play an important role in supporting their health and nutrition. Yet, little is known regarding the role of the host and environment in driving these communities. Here, bacterial assemblages were surveyed from seawater and gut aspirates of farmed *C. gigas* and co-occurring wild *M. galloprovincialis* in summer and winter using Illumina 16S rRNA gene sequencing. Unlike seawater, which was dominated by Pseudomonadata, bivalve samples largely consisted of Mycoplasmatota (Mollicutes) and accounted for >50% of the total OTU abundance. Despite large numbers of common (core) bacterial taxa, bivalve-specific species (OTUs) were also evident and predominantly associated with *Mycoplasmataceae* (notably *Mycoplasma*). An increase in diversity (though with varied taxonomic evenness) was observed in winter for both bivalves and was associated with changes in the abundance of core and bivalve-specific taxa, including several representing host-associated and environmental (free-living or particle-diet associated) organisms. Our findings highlight the contribution of the environment and the host in defining the composition of the gut microbiota in cohabiting, intergeneric bivalve populations.

**Supplementary Information:**

The online version contains supplementary material available at 10.1007/s00248-023-02192-z.

## Introduction

Microbes are ubiquitous and vital components of marine ecosystems that interact and form various, often intimate, relationships with an array of marine animal life [1]. Those associated with the gastrointestinal (GI) tract are considered to be of particular importance in supporting the animal’s health and nutrition and are driven by an array of both intrinsic factors, such as host physiology, genetics, age, growth, sex, immune status and life stage, and extrinsic factors, such as diet and environmental conditions [2, 3]. The GI tract of healthy animals is thought to harbour rich and diverse populations of both resident and transient microbes, of which bacteria are predominant constituents. During physiological and/or environmental stress, opportunistic microbes may outcompete resident populations for resources, leading to an imbalance in community composition, and possible immune suppression and the emergence of disease [4–6]. For sessile, suspension-feeding marine invertebrates, who actively ingest and are subject to local fluctuations in the surrounding environmental microbial consortia, host-microbe interactions and relationships, are likely to be of particular importance in supporting and influencing their ecology. However, unlike vertebrates, our understanding of marine invertebrate host-microbe interactions is limited [7], though is thought to be similarly shaped through co-evolutionary pressures, leading to the selection of species which support host health and metabolism [8].

While recent efforts have focused on the association and contributions of microbes belonging to particular invertebrate hosts (notably sponge or coral holobiont systems), our knowledge of other important marine invertebrate species like bivalves is gaining momentum due to their commercial significance and the tremendous risks posed by various pathogens [9–12]. Like in other marine animals, microbial community composition in bivalves varies across the different body regions, with the GI tract, gills, pallial fluid or haemolymph supporting distinct, tissue-specific assemblages [13–15]. Variations in the physicochemical characteristics and/or underlying immune functions within these regions are likely contributing features in the selection and enrichment of these assemblages [13, 15–17], which together are thought to drive the host phenotype as constituent members of the bivalve’s microbiome [11, 18, 19]. As reported for particular bivalves like oysters, these communities may, however, be influenced by particular stressors (e.g., elevated water temperatures), leading to microbiome imbalances that have the capacity to influence normal host functioning and susceptibility to pathogen infection [20–23]. This is of particular concern for oysters and other commercially important species like mussels, where the threat of a changing climate, seasonal mass mortality events and population decline is becoming increasingly apparent [24–27]. Our ability to gauge the magnitude that such risks represent though is largely dependent upon our understanding of the natural dynamics of the microbiome and the factors influencing its composition. This includes the role that host genetics plays in the selection of particular (core) constituents, their contribution to key host processes and the impact of environmental change at both a spatial and temporal scale. For most bivalve species, such knowledge currently remains limited, though it has the capacity to further support efforts that seek to use the microbiome as a predictive marker of environmental stress and disease susceptibility [28].

The Pacific oyster (*Crassostrea gigas*) and the Mediterranean mussel (*Mytilus galloprovincialis*) are two globally important species of significant economic value, accounting for up to ~30% of the world’s commercial bivalve production [29]. Like other bivalves, these species occupy bays, estuaries and near shore coastal waters and, at least in their native range, are also considered important for supporting the broader dynamics of marine ecosystems through the roles they play in nutrient cycling, habitat formation and modification and trophodynamics [30]. Having been introduced in other parts of the world like Australia through farming and early immigration [31], these species are able to translocate into and cohabit surrounding areas [32, 33] where they interact to compete for similar food sources [34]. In parts of the Northern Hemisphere, the invasion of *C. gigas* beds with mussel species (namely *M. edulis*) has led to the formation of ‘oyssel’ reef systems [35]. Alongside the valuable insights that this presents for elucidating the functioning of species assemblages [35], the cohabitation of bivalves also offers a prospect for delineating and exploring the role of host genetics and environment on the gut microbiome. However, while current insights from these species suggest a likely role for the host in the occurrence of select bacterial taxa (including the differential enrichment of potentially pathogenic *Vibrio* spp.) [36], little is known regarding the influence of seasonality on these communities.

Here, we aim to understand the influence of host species and environment on the gut microbiome (bacterial assemblages) of the intergeneric, cohabiting marine bivalves *C. gigas* (Ostreidae) and *M. galloprovincialis* (Mytilidae)*.* Specifically, comparative evaluations of the gut bacterial assemblages from farmed *C. gigas* and wild *M. galloprovincialis* collected from the same site in summer and winter were performed using an Illumina 16S rRNA gene sequencing approach. The impact of the surrounding environmental bacterial consortia on these gut bacterial communities were also evaluated by collecting and comparing samples obtained from seawater from the same site during summer and winter. Our study represents one of the first to investigate the differences in cohabiting bivalves between environmentally contrasting times of the year, where such knowledge could be used to further support our current understanding of host-specific microbiomes and the roles they play in supporting host ecology, and for inferring potentially disparate changes in health and disease that may arise from future stressors.

## Methods

### Field Sampling

Pacific oyster, Mediterranean mussel and seawater samples were collected from Coffin Bay, SA, Australia, at two time points in 2017, one in February and one in August 2017 (austral mid-summer and late winter, respectively) where the greatest disparity in mean monthly water temperatures is typically observed, recorded here as 18.6 ± 1.9°C (Feb) and 13.6 ± 0.6°C (Aug) (Table 1). The oysters were farmed using the longline method where four parallel lines were strung between wooden posts, and oysters were hung in plastic baskets (comprising up to ~120 individuals per basket). Oysters were collected from baskets graded for market size at the farm and likely represent mixed genetic cohorts, while wild mussels were randomly collected from the wooden posts at the same farm (where they were widely distributed over the sampling area). Seawater samples were collected at the farm at a depth of ~1 m using 2-L sterile glass bottles. In summer and winter, 30 Pacific oysters, 30 Mediterranean mussels and 3× 2 L seawater samples were collected (total = 60 Pacific oysters, 60 Mediterranean mussels and 6 seawater samples) (Table 1). All samples were stored at 4°C immediately upon collection and were transported to the laboratory at the Lincoln Marine Science Centre (Port Lincoln, SA, Australia) for further processing within 24 h of collection. Oysters and mussels were cleaned of fouling organisms (e.g. barnacles) and blotted with paper towel for weight measurements. Oysters and mussels of a comparable shell length of ~30–70 mm from the anterior to the posterior of shell were used in this study (as both bivalves are expected to reach this size range within the first 12 months of growth [37, 38]) and were absent of any obvious (symptomatic) features of disease. Downstream comparisons between bivalve species were conducted on matched samples (i.e. *small oysters* vs *small mussels* in summer, and *large oysters* vs *large mussels* in winter). In addition, due to the considerable variability observed in the mussel populations during the summer sampling period, a further comparison between mussels with different shell lengths was conducted to explore for possible size class differences.

**Table 1:** Samples collected and sequenced for bacterial community comparisons from Coffin Bay, South Australia. Monthly water temperatures (mean ± SD) are provided

Species	Oyster, *C. gigas*		Mussel, *M. galloprovincialis*	
Season	Summer (Feb)	Winter (Aug)	Summer (Feb)	Winter (Aug)
No. of bivalve samples collected/sequenced	30/30	30/30	30/30	30/26
Water samples (2 L)	3	3	3	3
Collection date	07.02.17	07.08.17	07.02.17	07.08.17
Water temp. (°C)^†^	18.6 ± 1.9	13.6 ± 0.6	18.6 ± 1.9	13.6 ± 0.6

^†^Average water temperatures were derived from data obtained from the Australian Ocean Data Network (AODN) Portal-Integrated Marine Observing System (IMOS) [https://imos.org.au/facilities/aodn]

Oysters and mussels were cleaned with 70% ethanol to minimise potential contamination arising from bacteria on the outer shell surfaces. Gut (stomach) contents from the oysters and mussels were collected by carefully prying open the shells and inserting a sterile glass pasture pipette fitted with a rubber bulb (Wheaton, DWK) through the mouth and applying gentle suction. The gut content from each individual (~200 μL) was dispensed into sterile cryovials and stored in liquid nitrogen for downstream DNA extraction. Gut and water samples were then transported to the Molecular Science Laboratory at South Australian Research and Development Institute (West Beach, SA, Australia) under temperature-controlled conditions for downstream analysis.

### DNA Extraction from Bivalve and Seawater Samples

DNA was extracted from bivalve gut aspirate samples using the FastDNA^TM^ spin kit for soil (MP Biomedicals) according to the manufacturer’s instructions. Seawater samples were also extracted using the same kit but were first filtered using Nalgene™ Rapid-Flow™ filter units with a sterile disposable bottle on the top (filter capacity 500 mL, pore size 0.2 μm, 45-mm bottle neck, Sigma®), and the filter paper was cut into pieces and placed within the accompanying lysing matrix tubes. All DNA samples were concentrated by ethanol precipitation using standard procedures, quantified using the NanoDrop 2000 spectrophotometer (Thermo Fisher Scientific) and stored at −20°C until downstream library preparation.

### PCR Amplification, Library Preparation and Sequencing

The V1–V2 hypervariable region of the 16S rRNA gene was amplified from DNA extracts using a multi-step PCR procedure as developed and implemented elsewhere with eubacterial primers 27F and 338R [39, 40]. Specifically, for library generation, 25 ng of sample DNA was subjected to an initial 20 cycles of PCR comprising 2.5 mM deoxynucleoside triphosphates, 2.5 U μL^−1^ PrimeSTAR® HS DNA Polymerase (Takara Bio), 5× PrimeSTAR® Buffer (Takara Bio) and 10 μM of each primer, with cycling consisting of initial denaturation at 95°C for 3 min, followed by consecutive rounds of 98°C for 10 s, 55°C for 10 s and 72°C for 45 s. One microliter from this reaction was used as template for a further 15 cycles of PCR (using the same conditions and cycling parameters) for incorporating individual 6 nt barcodes and Illumina-specific adaptors. A final 10 cycles of PCR were conducted using 1 μL from this second reaction for incorporating the Illumina multiplexing sequencing and indexing primers. PCR products were visualised by agarose gel electrophoresis and those of the expected size (~438 bp) were subsequently purified using Agencourt AMPure XP beads (Beckman Coulter) and quantified using the Quant-iT™ PicoGreen® dsDNA kit (Life Technologies). Amplicons were pooled in equimolar ratios and sequenced on the Illumina MiSeq platform (Illumina) using 250 nt paired-end sequencing chemistry through the Australian Genome Research Facility (AGRF, North Melbourne, VIC, Australia). Amplicons obtained from gDNA extracts of *Lactobacillus reuteri* were sequenced alongside the samples as a control.

### Bioinformatics Analysis

A total of ~12.5 million raw sequence reads were obtained from a total of 122 samples (*n*=60/60 oyster; *n*=56/60 mussel; *n*=6/6 seawater). Reads were assembled by aligning the forward and reverse reads using PEAR (version 0.9.5) [41], and the primers were identified and trimmed. Trimmed sequences were processed using the Quantitative Insights into Microbial Ecology (QIIME) (version 1.8) [42], USEARCH (version 8.0.1623) [43] and UPARSE software [44]. Using USEARCH tools, sequences were quality filtered to remove low-quality reads, full-length duplicate sequences and singletons. Sequences were clustered into operational taxonomic units (OTUs) at a minimum identity of 97%, with putative chimaeras removed using the RDP-gold database as a reference [45].

A total of 5,517,945 high-quality, paired-end reads (mean = 45,229 ± 17,036 reads/sample; min=18,427; max=112,618) were clustered into 22,402 OTUs. These OTUs were further filtered as conducted previously [40] where only those that contributed to > 0.01% of the bivalve-associated (*n*=116) or > 0.01% of the seawater dataset (*n*=6) were retained. The resultant OTUs were interrogated using the SeqMatch function of the RDP database [46] as well as SILVA [47], whereby taxonomic lineages based on the SILVA taxonomy and the best hit from RDP were assigned for each OTU. Those OTUs representing chloroplast or fungi were removed from the dataset, leaving a total of 659 OTUs for downstream analysis. Rarefaction curves were used to assess (retrospectively) sampling depth (Supplementary Fig. 1).

### Statistical Analysis

The final dataset comprised 659 OTUs from 122 samples (60 oyster gut, 56 mussel gut and 6 seawater samples) and were used for statistical analysis using Primer-E version 7.0.11 [48]. Non-metric multidimensional scaling (nMDS) ordination plots were generated to visualise the global bacterial community structures from these samples using Bray-Curtis similarity resemblance [48, 49]. Bubble overlays were incorporated in the ordination plot comparing mussels and oysters to indicate variations in weight. Two-way and one-way permutational multivariate analysis of variance, PERMANOVA, was used to assess differences between groups of samples such as *oyster* vs *mussel*, *summer sampling month* (Feb) vs *winter sampling month* (Aug), *species* vs *sampling month* and *large* vs *small mussels* (based on groups of individuals with shell lengths > 60mm and < 40mm, respectively) allowing for type III (partial) sums of squares and fixed effects of sum to 0 for mixed terms. The *p*-values were generated using unrestricted permutations of raw data [48, 50] and were considered significantly different if *p* <0.05. Multivariate dispersion indices (MVDISP, IMD routines) were calculated in Primer-E to gauge the degree of inter-individual variation within and between sample groups. Diversity measures for each group of samples were generated as box plots in Primer-E and included species/OTU richness (S), Pielou’s evenness (J’), Shannon index (H’), Simpson index (1-λ), average taxonomic distinctness (avTD) (delta+) and variation in taxonomic distinctness (varTD) (lambda+). The latter two measures are respectively used to gauge the average taxonomic distance between all pairs of species (as an indicator of the taxonomic breadth of the OTUs) and how consistently each taxonomic lineage is represented (as an indicator of the taxonomic evenness of the OTUs) [51, 52]. When comparing differences in the diversity indices between *small* and *large mussels*, an independent *t*-test was performed, while for comparisons between bivalve *species* and *season*, a two-way crossed ANOVA was used, where alpha was set to 0.05 (GraphPad Prism, version 8.1.1). In both cases, distribution (normality) was first assessed using the D’Agostino and Pearson and the Shapiro-Wilk algorithms. Variations in the abundance of bacterial taxa were visualised using stacked bar charts in Primer-E, with Venn diagrams used to display the numbers of shared (and likely core) and unique OTUs among oyster, mussel and seawater samples. Differential abundance analysis based on linear discriminant analysis (LDA) effect size (LEfSe) was conducted in MicrobiomeAnalyst [53, 54] to discern the significant classes, families and/or OTUs contributing to the observed differences among treatments; as determined using the Kruskal-Wallis rank test (unadjusted/adjusted *p*-value cut-off = 0.01), with the Log LDA score value set to 2.0 and significant taxa/OTUs given in descending order from the highest to lowest LDA score. Univariate measures of shell length and weight were visualised using a scatter plot in Primer-E.

## Results

 Comparison of length and weight measurements from individuals provided size class information that was used to infer potential cohort differences. Distinct size classes were observed for both oysters and mussels between the sampling periods (Supplementary Fig. 2). In the *summer* sampling month, oysters comprised smaller shell lengths and weights (mean 45.9 ± SD 3.6 mm; mean 35.2 ± SD 1.9 g), while in the *winter* sampling month, they had larger mean shell lengths and weights (64.9 ± 3.9 mm; 52.3 ± 10.5 g). Similar differences were also observed for mussels, though those collected in summer appeared to consist of at least two separate size classes (and thus likely cohorts): one group with *smaller* shell lengths of <40 mm (18 mussels: 33.6 ± 2.8 mm; 16.5 ± 4.0 g) and one with *larger* shell lengths of >60 mm (12 mussels: 73.0 ± 12.0 mm; 83.8 ± 33.5 g). Mussels in winter comprised a mean shell length of 64.0 ± 3.2 mm and a mean weight of 20.5 ± 3.3 g.

**Fig. 1:**
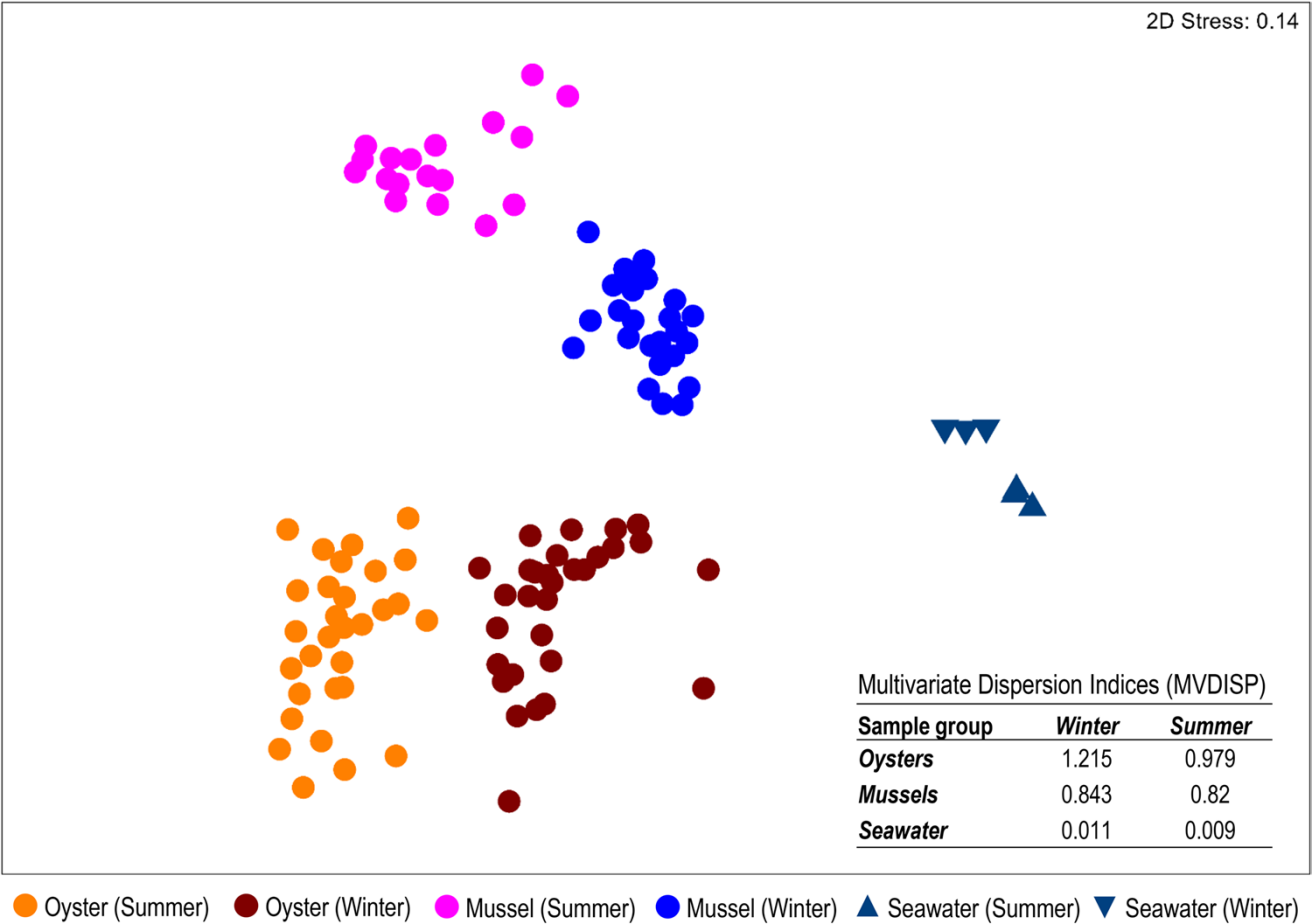
Ordination plot depicting the global differences in the bacterial community composition between matched oyster and mussel gut and seawater samples collected in summer and winter from Coffin Bay (South Australia), as assessed by non-metric multidimensional scaling (nMDS) using Bray-Curtis dissimilarity. Corresponding multivariate dispersion indices (MVDISP) representing the global variation in the bacterial community composition among samples are given for each sample group in summer and winter (inset table), where higher values represent greater variation and lower values less variation among samples

The bacterial communities of farmed oysters and cohabiting wild mussels were surveyed from aspirated gut contents, as well as from six seawater samples obtained from the same farm site (3 per sampling month). Of the total of 659 OTUs obtained for analysis, 105 were unique to bivalves, 15 to seawater and 539 that were shared between bivalves and seawater (Supplementary Datasheet 1). Despite the large number of shared OTUs, ordination of the samples revealed that the bivalve samples clustered independently to those obtained from seawater (Fig. 1). Furthermore, samples from oysters and mussels clustered independently of one another and in association with the sampling month in which they were obtained (i.e., austral summer or winter). This observation was confirmed by two-way PERMANOVA (pseudo-*F* = 66.31, *p*-value = 0.0001; pseudo-*F* = 40.92, *p*-value = 0.0001, respectively). However, there was a significant interaction effect between *species* and *sampling month* (pseudo-*F* = 18.74, *p*-value = 0.0001), indicating that changes between the summer and winter sampling months were species-specific. This was accompanied by notable differences in the calculated multivariate measures of dispersion (MVDISP), with the greatest variation among individuals within sample groups (as indicated by a higher MVDISP value) observed for *oysters* compared to *mussels* (independent of sampling month) (Fig. 1, inset table). Variation among individual oyster samples (and to a lesser extent mussels and seawater) also appeared to be more pronounced in *winter* compared to *summer*.

**Fig. 2:**
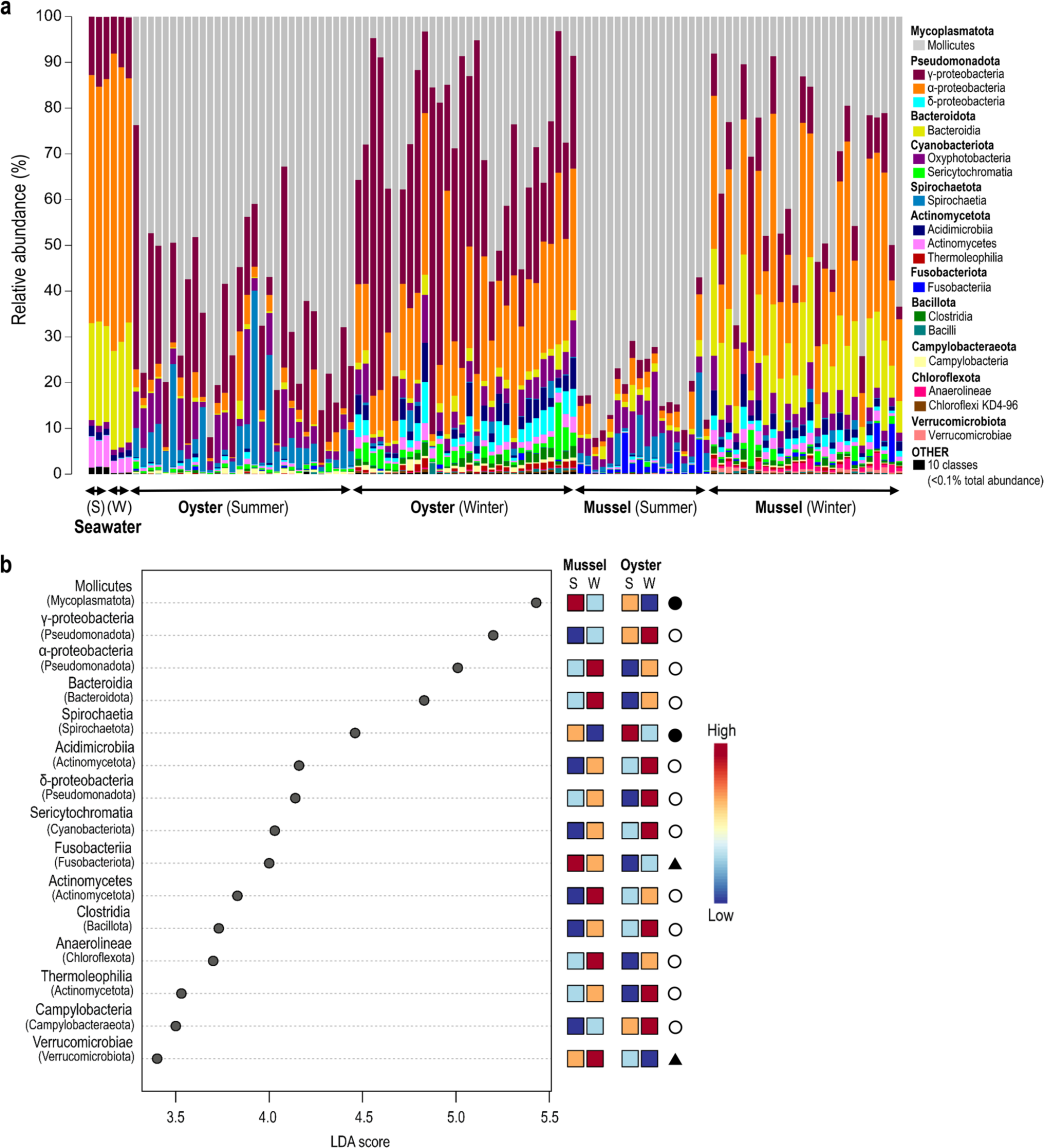
Mean relative abundances of bacterial classes (**a**) associated with seawater and matched oyster and mussel gut samples collected in summer (S) and winter (W) from Coffin Bay, South Australia; and linear discriminant analysis (LDA) effect size (LEfSe) plot (**b**) displaying the differentially abundant bacterial classes associated with the matched summer and winter oyster and mussel gut samples. Differentially abundant features were determined using the Kruskal-Wallis rank test (adjusted *p*-value [FDR] cut-off = 0.01), with the Log LDA score value adjusted to 2.0 and significant taxa given in descending order from the highest to lowest LDA score. The heat key denotes the rank-ordered abundance of each class. Symbols represent classes that occurred in higher abundance in both bivalves in summer (black circle) or in winter (white circle) or which had notably disparate abundances between bivalves in the summer and winter sampling months (black triangle)

In evaluating all samples, OTUs represented bacterial taxa belonging to 17 phyla, 28 classes, 90 orders, 150 families and 285 genera, of which the two phyla Mycoplasmatota and Pseudomonadota accounted for >80% of the total OTU abundance (Fig. 2a). Unlike seawater, which was dominated by α- and γ-proteobacteria and to a lesser extent Bacteroidota (Bacteroidia) and Actinomycetota (Acidimicrobiia and Actinomycetes), samples from bivalves largely consisted of Mycoplasmatota (Mollicutes) as well as α-, γ- and δ-proteobacteria, Spirochaetota (Spirochaetia) and Cyanobacteriota (Oxyphotobacteria and Sericytochromatia). The Mycoplasmatota (Mollicutes) were largely associated with bivalve samples, accounting for ~52% of the total OTU abundance, as derived from a total of 36 OTUs, two most closely representing *Spiroplasmataceae* (<72% identity), which were almost exclusively associated with mussels, and 34 most closely representing *Mycoplasmataceae* (<83% identity), of which 31 were shared between both bivalve species (Supplementary Datasheet 1). The greatest proportion of Mycoplasmatota occurred in summer for both oysters (mean 64.2 ± SD 16.9%) and mussels (80.9 ± 8.7%). In contrast, the phyla Pseudomonadota had a lower mean abundance in *summer* compared to *winter* for both oysters (21.3 ± 12.5% vs 56.7 ± 18.4%) and mussels (6.3 ± 3.0% vs 36.0 ± 12.1%). These findings were supported by differential abundance analysis (as determined using the Kruskal-Wallis rank test, adjusted *p*-value cut-off = 0.01), which also revealed a *summer* vs *winter* trend in the proportions of other major taxonomic groups, including a higher abundance of Spirochaetota in both bivalves in summer and, conversely, a higher proportion of Bacteroidota, Actinomycetota (Acidimicrobiia, Actinomycetes and Thermoleophilia), Cyanobacteriota (Sericytochromatia), Bacillota (Clostridia), Chloroflexota (*Anaerolineae*) and Campylobacteraeota (Campylobacteria) in winter (Fig. 2b, Supplementary Table 1). Two groups, however, appeared to have disparate abundances between the *summer* and *winter* sampling months, with Fusobacteriota more abundant in summer in mussels and in winter in Oysters and Verrucomicrobiota in winter in mussels and in summer in oysters. The 10 most dominant bivalve associated OTUs accounted for >50% of the total standardised sequence reads and included taxa largely related to Mollicutes, including *Mycoplasma* spp. (OTU 7, mean abundance of 9.2%; OTU 6, 6.4%; OTU 4, 4.8%; OTU 51, 3.3%; OTU 19, 3.1%), *Candidatus Bacilloplasma* sp. (OTU 11, 5.1%) and uncultured *Mycoplasmataceae* spp. (OTU 2, 3.9%; OTU 14, 3.9%; OTU 17, 2.6%), as well as the γ-proteobacteria *Halioglobus* sp. (OTU 1, 8.9%) (Supplementary Datasheet 1).

### Defining a Role for Host Species in Gut Bacterial Community Composition in Bivalves

**Fig. 3:**
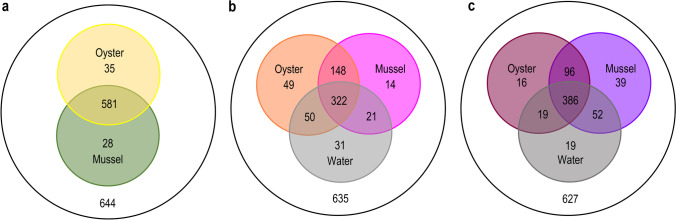
Venn diagrams indicating the distribution of unique and shared bacterial OTUs in **a** oyster and mussel gut, irrespective of the summer or winter month of sampling; **b** oyster and mussel gut and seawater in summer; and **c** oyster and mussel gut and seawater in winter. Values inside the outermost circles indicate total number of observed OTUs

To understand the influence of host species on bivalve gut bacterial communities, core (shared) and unique bacterial constituents were first evaluated from comparisons between all samples (irrespective of sampling month). Of the 644 OTUs that were detected from bivalves, only 35 were unique to oysters and 28 to mussels, with the majority (~90%) being shared (Fig. 3a). Of these, 13 OTUs from oysters and seven OTUs from mussels were not detected (or occurred in very low abundance) in seawater. The top three most prevalent in oysters are related to taxa belonging to *Anaplasmataceae* (α-proteobacteria) (OTU 140, *Candidatus* Neoehrlichia, min. 0–max. 0.2%), *Spirochaetaceae* (Spirochaetota) (OTU 263, *Spirochaeta* 2, 0–1.9%) and *Mycoplasmataceae* (Mollicutes) (OTU 9073, *Mycoplasma* sp., 0–1.4%) (Supplementary Table 2), while in mussels, the most prevalent were *Mycoplasmataceae* (OTU 115, *Mycoplasma* sp., min. 0–max. 3.6%; OTU 261, *Mycoplasma* sp., 0–1.5%) and *Spiroplasmataceae* (OTU 180, *Spiroplasma* sp., 0–1.4%) (Supplementary Table 3). Alongside this, in assessing the differentially abundant families and OTUs associated with these samples, certain taxa also appeared to be preferentially more abundant in one of the two bivalve species—as determined using the Kruskal-Wallis rank test (adjusted *p*-value cut-off = 0.01) (Supplementary Fig. 3a and b). In oysters, this included a total of 7 families, including the γ- and α-proteobacteria families *Halieaceae* (OTU 1, *Halioglobus* sp.), *Kiloniellaceae* (OTU 22, *Kiloniella* sp.) and *Pseudomonadaceae* (OTU 16, *Pseudomonas alcaligenes*), as well as an unclassified *Sericytochromatia* (Cyanobacteriota), Campylobacteraeota families *Helicobacteracea*e and *Campylobacteraceae* and a number of other OTUs belonging to *Mycoplasma*/uncultured *Mycoplasmataceae* (OTUs 6, 4, 2, 13, 1474, 281, 3 and 27) and *Spirochaetaceae* (OTU 26, *Salinispira* sp.; OTU 38, uncultured *Spirochaetaceae*). In contrast, taxa belonging to 18 different families appeared to contribute to the differences observed for mussels. The most notable of these included *Flavobacteriaceae* (OTU 71, *Polaribacter* sp.; OTU 41, *Ulvibacter* sp.), *Rhodobacteraceae* (OTU 59, *Sulfitobacter* sp.; OTU 43, *Planktomarina* sp.) and *Fusobacteriaceae* (OTU 37, *Psychrilyobacter* sp.). In addition, like that observed for oysters, a number of OTUs belonging to the *Mycoplasmataceae* and *Spirochaetaceae* also appeared to contribute to the observed differences, including OTU 11 (*Candidatus* Bacilloplasma sp.), OTU 14 and 17 (uncultured *Mycoplasmataceae*), OTU 51 (*Mycoplasma* sp.) and OTU 28 (*Spirochaeta* 2).

**Fig. 4:**
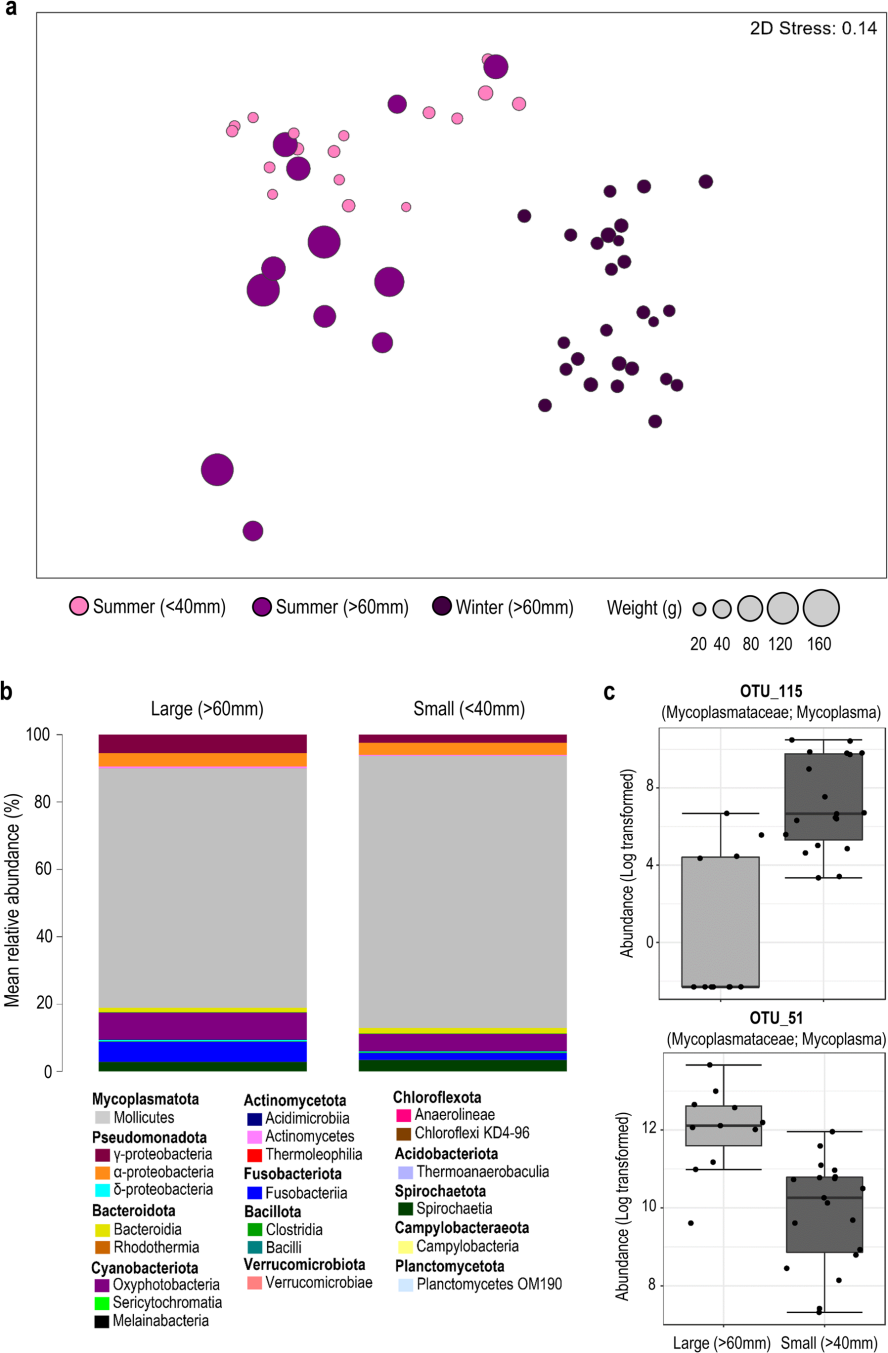
Comparison of the bacterial compositional differences between gut samples obtained from mussels with large (>60mm) and small (<40mm) shell lengths. **a** Global differences in the bacterial community composition between large and small mussels collected in summer (and in comparison to large mussels in winter), as assessed by non-metric multidimensional scaling (nMDS) using Bray-Curtis dissimilarity. Bubble overlays represent mussel weight (g). **b** Mean relative abundance of bacterial classes from large and small summer mussel samples and **c** differentially abundant OTUs observed from large and small summer mussel samples as determined using the Kruskal-Wallis rank test (adjusted *p*-value [FDR] cut-off = 0.01)

Ordination of samples from mussels obtained in *summer* with large (>60 mm) and small (<40mm) shell lengths, and in comparison, to samples from mussels in *winter* with large shell lengths (>60mm), revealed the independent clustering and likely differences in the global bacterial community compositions of samples belonging to these size classes (Fig. 4a). This observation was confirmed by one-way PERMANOVA, revealing a significant difference between the large and small summer mussels (pseudo-*F* = 4.5604, *p*-value = 0.0028). No significant differences were observed, however, between these groups for measures of species/OTU richness (*p*-value = 0.2253; large mussels: mean 244 ± SD 48, small mussels: 263 ± 37), Shannon diversity (*p*-value = 0.6574; large mussels: 2.13 ± 0.59, small mussels: 2.04 ± 0.48) and Pielou’s evenness (*p*-value = 0.5610; large mussels: 0.39 ± 0.10, small mussels: 0.37 ± 0.09). Furthermore, though some (albeit slight) changes were observed in the mean abundances of various bacterial classes (Fig. 4b), differential abundance analysis revealed the occurrence of only five significantly different OTUs—as determined using the Kruskal-Wallis rank test (unadjusted *p*-value cut-off = 0.01) (Supplementary Table 4). Based on Log LDA scores, the two with the largest effect size included those most closely related to *Mycoplasma* spp.—OTU 115 (LDA -4.99) which had a higher abundance in samples from small mussels and OTU 51 (LDA 3.48) which had a higher abundance in samples from large mussels (Fig. 4c).

### Environmental Drivers of Gut Bacterial Community Composition in Bivalves

**Fig. 5:**
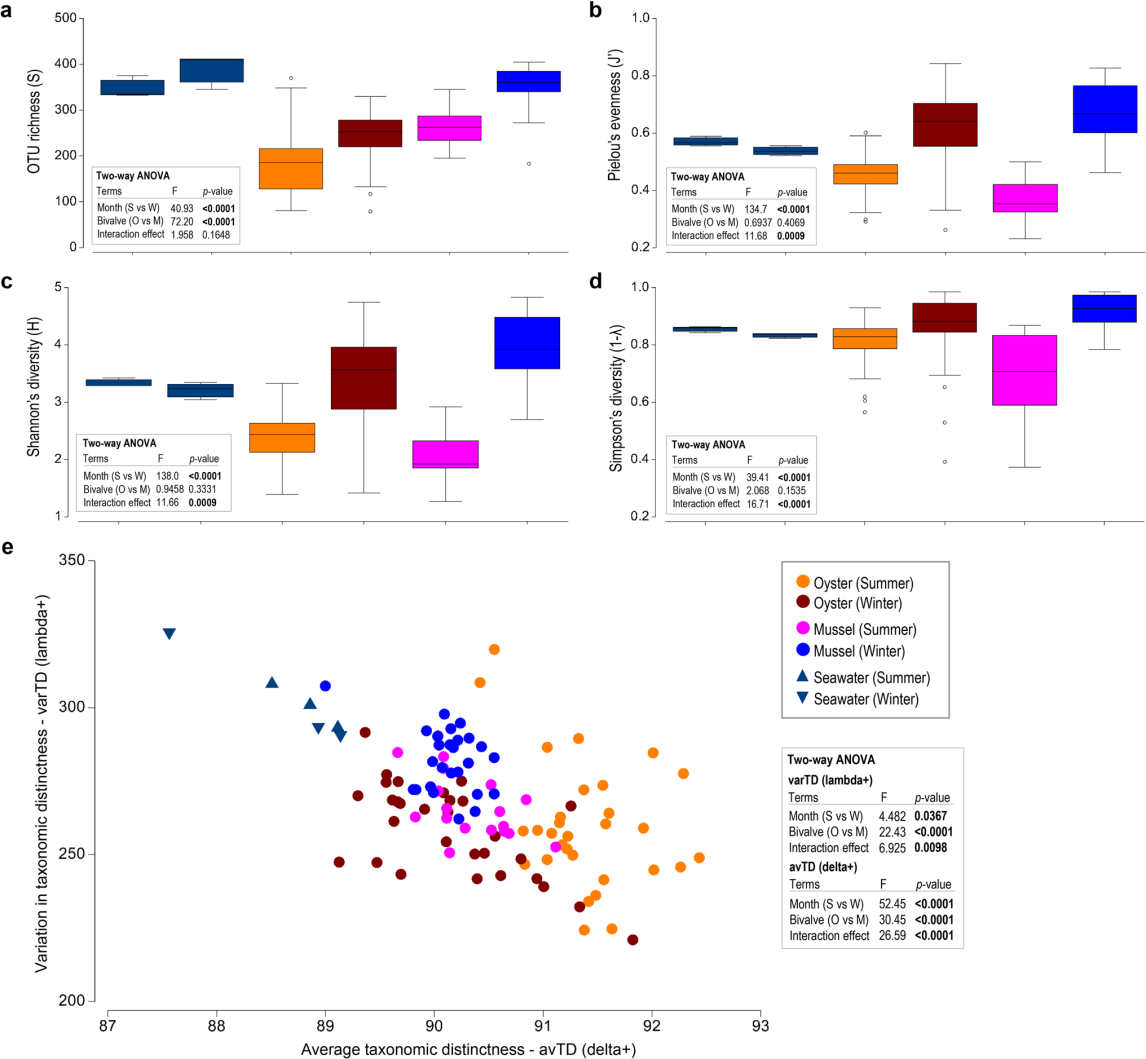
Measures of bacterial diversity from oyster and mussel gut, and seawater samples in summer and winter. Box plots represent the median, interquartile ranges (IQR) and distribution of measures of **a** OTU richness; **b** Pielou’s evenness; and **c**, **d** Shannon’s and Simpson’s indices of diversity. **e** Scatter plot charting the average taxonomic distinctness (avTD, delta+) as a function of variation in taxonomic distinctness (varTD, lambda+)

In exploring the impact of the surrounding environment (seawater) on the gut bacterial communities from bivalves, where the average monthly water temperature varied by ~5°C (Table 1), notable differences between the sampling periods were observed. Overall, while samples from mussels comprised a greater number of OTUs compared to oysters in both summer (263 ± 37 vs 184 ± 66) and winter (349 ± 46 vs 240 ± 60) (Fig. 5a), changes in species/OTU richness and diversity (Shannon and Simpson’s diversity and Pielou’s evenness) were apparent for both bivalves between the two sampling months. Most notably was a marked increase in these measures in winter (Fig. 5a–d). This observation was confirmed by two-way ANOVA, which crossed bivalve *species* with *sampling month*, revealing highly significant differences between summer and winter (*p*-value < 0.0001). However, there was a significant interaction effect between *species* and *sampling month* (*p*-value < 0.0001) for all measures except for species/OTU richness, indicating that while a similar increase in the number of OTUs occurred for both oysters and mussels in winter, there were likely species-specific differences in the types and/or relative abundances of these OTUs. A similar trend for measures of richness and diversity was also observed for the seawater samples between summer and winter, though the differences were not significant (*p*-value > 0.05). In evaluating the breadth and evenness of the taxonomic diversity of the OTUs within each sample (as assessed by comparing variation in taxonomic distinctness with average taxonomic distinctness), a significant difference was observed between bivalve species and sampling month (Fig. 5e). Samples from oysters typically comprised OTUs covering a greater breadth of taxa in summer compared to winter (based on a higher mean value for delta+: 91.39 ± SD 0.49 vs 90.16 ± 0.67 respectively), though were similarly evenly distributed across taxonomic lineages in both sampling months (based on similarly low mean values for lambda+: 259.97 ± 21.98 and 258.29 ± 15.72, respectively). In contrast, samples from mussels comprised OTUs covering a similar breadth of taxa in both summer and winter (based on similar mean values for delta+: 90.33 ± 0.38 and 90.12 ± 0.31, respectively), though were more unevenly distributed across taxonomic lineages in winter compared to summer (based on a higher mean value for lambda+: winter 282.27 ± 10.84 vs summer 266.81 ± SD 11.50). This observation was supported by the occurrence of a significant interaction effect between *species* and *sampling month* (for both measures of delta+ and lambda+), indicating that changes between the summer and winter sampling months were species-specific. Despite seawater samples comprising the greatest number of OTUs (Fig. 5a), these OTUs represented a substantially lower breadth of taxa and were more unevenly distributed across taxonomic lineages in both summer and winter compared to those from bivalves (based on lower values for delta+ and higher values for lambda+) (Fig. 5e).

**Fig. 6:**
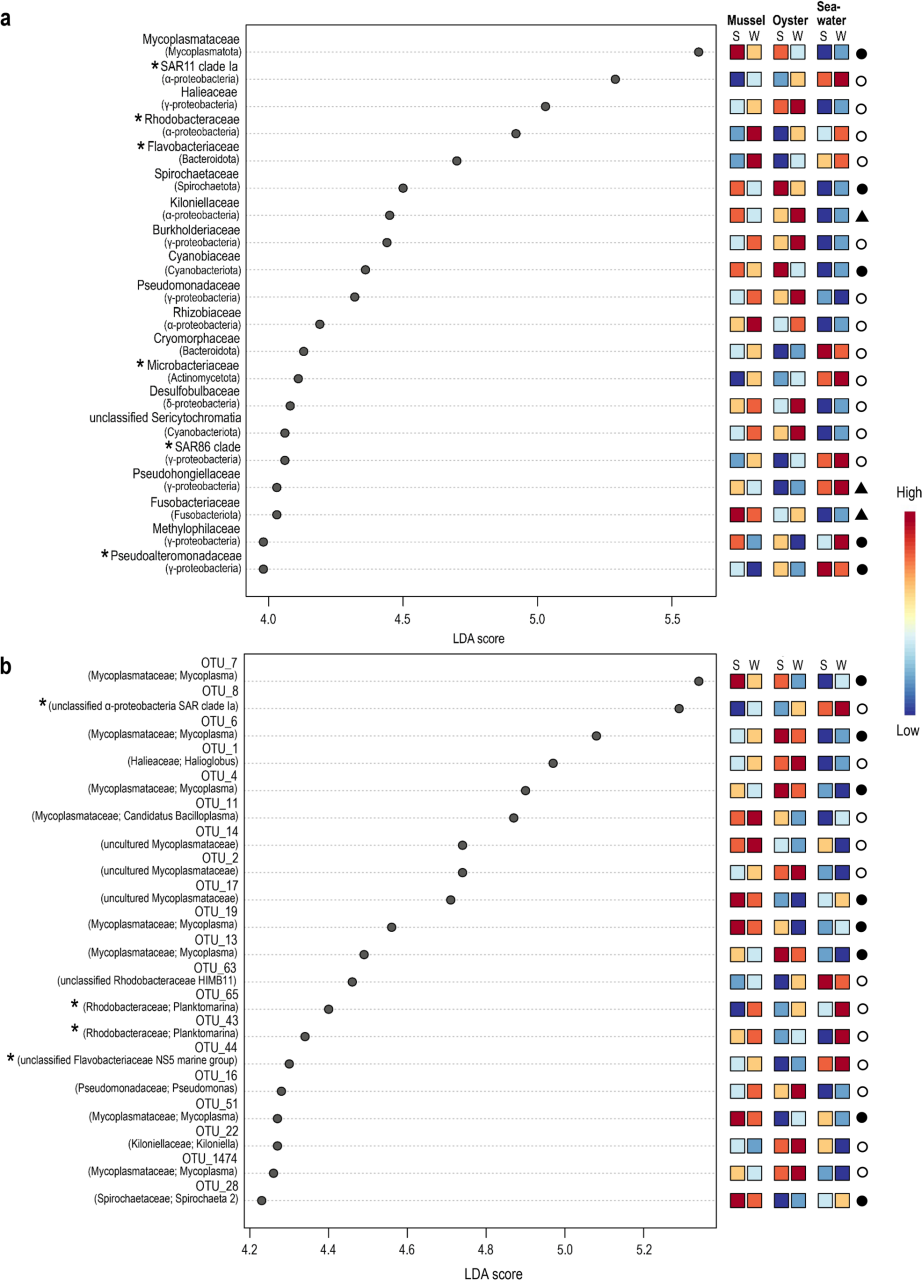
Linear discriminant analysis (LDA) effect size (LEfSe) plots displaying the top 20 differentially abundant bacterial families (**a**) and OTUs (**b**) from mussel and oyster gut and seawater samples obtained in summer (S) and winter (W) from Coffin Bay, South Australia. Differentially abundant features were determined using the Kruskal-Wallis rank test (adjusted *p*-value [FDR] cut-off = 0.01), with the Log LDA score value adjusted to 2.0 and significant taxa given in descending order from the highest to lowest LDA score. The heat key denotes the rank-ordered abundance of each taxa. Symbols represent taxa with the highest abundance in both bivalves in summer (black circle) or in winter (white circle) or which had notably disparate abundances between bivalves in the summer and winter sampling months (black triangle). Taxa marked with an asterisk represent those that had a corresponding high seasonal abundance in seawater

Differential abundance analysis comparing oyster and mussel gut and seawater samples in *summer* and *winter* revealed a total of 29 families and 151 OTUs that were significantly different (as determined using the Kruskal-Wallis rank test, adjusted *p*-value cut-off = 0.01). In evaluating the 20 most differentially abundant families and OTUs with the greatest effect size (based on the Log LDA scores), distinct patterns were observed between the sampling months whereby a concomitant increase in the abundance of certain taxa was observed in either summer or winter in both bivalve species (Fig. 6, Supplementary Table 5 and 6). This included 5 families in summer (*Mycoplasmataceae*, *Spirochaetaceae*, *Cyanobiaceae* (*Synechococcus* and *Cyanobium* spp.), *Methylophilaceae* (OM43 clade) and *Pseudoalteromonadaceae* (*Pseudoalteromonas* spp.)) and 12 in winter (α-proteobacteria SAR11 clade 1a, *Halieaceae*, *Rhodobacteraceae*, *Flavobacteriaceae*, *Burkholderiaceae*, *Pseudomonadaceae*, *Rhizobiaceae*, *Cryomorphaceae*, *Microbacteriaceae*, *Desulfobulbaceae*, unclassified Sericytochromatia and γ-proteobacteria SAR86 clade). Of these, six also had an associated high abundance in the seawater in either the summer or winter sampling months. This included *Pseudoaltermonadaceae* in summer and α-proteobacteria SAR11 clade 1a (OTU 8), *Rhodobacteraceae* (OTU 43 and 65, *Planktomarina* spp.), *Flavobacteriaceae* (OTU 44, unclassified NS5 marine group) and γ-proteobacteria SAR86 clade in winter (as marked by asterisks in Fig. 6a and b). The majority of OTUs contributing to the observed differences between the summer and winter sampling months included those most closely related to members of the *Mycoplasmataceae* (notably *Mycoplasma*), whereby in mussels, OTUs 7, 17, 19 and 51 were more abundant in summer, and OTUs 11 (*Candidatus* Bacilloplasma sp.) and 14 (*Mycoplasma* sp.) were more abundant in winter. A similar trend was observed for *Mycoplasmataceae* related taxa in oysters, whereby OTUs 6, 4 and 13 were more abundant in summer and OTUs 2 and 1474 in winter. Other OTUs with a notable increase in abundance in either the summer or winter sampling months included OTU 1 (*Halioglobus* sp. 79.46% identity), OTU 16 (*Pseudomonas pseudoalcaligenes*, 99.08% identity) and OTU 22 (*Kiloniella* sp., 80.33% identity) in oysters in winter and OTU 28 (*Spirochaeta* 2 sp., 77.24% identity) in mussels in summer (Fig. 6b, Supplementary Table 6 and Supplementary Datasheet 1).

## Discussion

The gut bacterial assemblages of two intergeneric cohabiting marine bivalves *Crassostrea gigas* and *Mytilus galloprovincialis* in summer and winter were revealed. Though each harboured distinct communities that differed to that of the surrounding environment, a large number of common (core) bacterial OTUs were observed between bivalves, suggesting a role of both the environment and the host in determining the bacterial community composition of the gut. Similar findings have been reported elsewhere in comparative studies of the haemolymph and digestive gland of *C. gigas* and *M. galloprovincialis* [36], as well as the gut of the eastern oyster (*Crassostrea virginica) and blue mussel (Mytilus edulis)* [55], and are thought to occur as a result of the ingestion of common planktonic or aggregate-associated environmental consortia through filter feeding [56]. Here, OTUs typically representing environmental taxa such as the γ-proteobacteria family *Halieaceae* (namely *Halioglobus* sp.) [57] and Cyanobacteriota (which are likely to be fed upon by bivalves [55]) were found to occur in the gut aspirates of both bivalves and thus likely reflect such constituents. However, other likely environmental associated OTUs were also observed though were found to be more predominant in either oysters or mussels. Specifically, alongside *Halioglobus*, the α-proteobacteria family *Kiloniellaceae* (*Kiloniella* sp.) and the non-photosynthetic Cyanobacteriota *Sericytochromatia* were more abundant in oysters, while *Flavobacteriaceae* (namely *Polaribacter* and *Ulvibacte*r spp.) as well as *Rhodobacteraceae* (*Planktomarina* and *Sulfitobacter* spp.) and *Fusobacteriaceae* (*Psychrilyobacter* sp.) were more abundant in mussels. Furthermore, as also reported for other mytilids [55], a greater number of OTUs were found to occur in samples from mussels compared to oysters between the summer and winter sampling months. Given the varied spectrum of particle sizes that are selectively fed upon by these bivalves (where *M. galloprovincialis* is able to access a wider range of food particle sizes than *C. gigas*) [58] and the accompanied varied microbial diversity associated with such particles (where richness increases with particle size) [59], such a finding likely reflects variations in host feeding ecology and the types of bacteria that are thus introduced into the gut. The prevalence of OTUs in mussels representing particular organisms like *Rhodobacteraceae* which have been reported as constituents of larger particles sizes [59], as well as *Psychrilyobacter* spp. which are among some of the most significant degraders of detrital matter [60], may further support the influence that particle size has on gut microbiota composition. However, whether such organisms are directly or indirectly selected as components of the diet for enabling their cohabitation through, e.g. resource partitioning (as suggested for freshwater mussels [61]), or are later excreted (as transient populations) within the pseudofaeces or taken up as resident components of the microbiota requires further investigation. With certain taxa like Cyanobacteriota also having been suggested to play a role in reducing susceptibility to disease in oysters (possibly as endosymbionts) [28, 62], establishing the relevance of such organisms as components of the gut microbiota would be of considerable value.

Of particular importance in this study was the occurrence of key groups of bacteria that occurred predominantly in association with bivalve rather than seawater samples. Specifically, unlike seawater, which comprised larger proportions of OTUs associated with α- and γ-proteobacteria as well as Bacteroidota and Actinomycetota, more than half of the OTUs derived from bivalve samples appeared to be exclusively associated with Mollicutes (notably members of the family *Mycoplasmataceae* as well as *Spiroplasmataceae* and *Candidatus* Bacilloplasma). Though *Candidatus* Bacilloplasma was originally reported from the hindgut of terrestrial isopods [63], this and other members of the *Mycoplasmataceae* (particularly *Mycoplasma* spp.) have been detected and may represent key gut constituents in other marine organisms including in oysters and mussels [21, 55, 64–69]. In highlighting the utility of the gut aspiration-based approach for also surveying the likely resident bacterial populations, bacteria belonging to the class Mollicutes are generally considered to be host-associated (parasitic) organisms which, having undergone substantial reductive evolution, lack cell walls and have become reliant upon the host for supporting their metabolic processes [70]. The prevalence of certain taxa such as *Mycoplasma* in marine animals (particularly in fish) [71, 72] and the occurrence of several pathogenic species [9] has attracted considerable attention, though recent studies point towards a more mutualistic relationship. As inferred from metagenome-assembled genomes (MAGs) of Mollicutes associated with the gut of the eastern oyster (*C. virginica*), it was reported that such organisms (as being most closely related to *Mycoplasma* spp.) may also confer a benefit to the host by reducing parasite infection through the competitive sequestration of arginine [69]. However, given that increased abundances of particular Mollicutes-related taxa like members of the *Mycoplasmataceae* have also been observed to occur in oysters that are more susceptible to disease (namely in Pacific Oyster Mortality Syndrome, POMS) [28], their roles here in farmed oysters and cohabiting mussels require further examination. This should be extended to include other taxa like *Helicobacteraceae* and *Campylobacteraceae* whose roles in the bivalve host, to the best of our knowledge, remain unclear and may represent potential foodborne pathogens and/or environmental indicator organisms of human faecal pollution [73, 74].

That Mollicutes may, in particular, be relevant in the bivalve host was further evident here from the recovery of sequences belonging to a considerable number of related OTUs. Specifically, a total of 38 Mollicutes OTUs were detected from the bivalve gut samples, of which 36 were most closely related to *Mycoplasmataceae* (*Mycoplasma* spp.), with the majority (31) being shared between the two bivalve species. Interestingly, similarly diverse populations of Mollicutes-related taxa have also been previously reported for oysters (namely *C. virginica*), with a total of 36 distinct amplicon sequence variants (ASVs) found to belong to four major clades based on groups of environmental 16S rRNA gene reference sequences [72]. While *Mycoplasma* spp. are generally considered to have established unique relationships with individual hosts over extended periods of coevolution, they are thought to have descended from multiple bacterial lineages (rather than a single common ancestor) and appear to undergo rapid and divergent evolution, allowing them to rapidly adapt to changing microenvironments [75, 76]. With mussels (Family Mytilidae) and oysters (Family Ostreidae) having likely evolved at different times from a common ancestor [77], the detection of shared, diverse *Mycoplasmataceae*-associated OTUs is thus not surprising, and given their low sequence identities (~72-83%) may represent different (and likely novel) species that have emerged from multiple bacterial lineages within the gut of these bivalves. Of course, more detailed, targeted analyses need to be conducted to verify the diversity of these organisms, notably to discount biases that may arise from the presence of pseudogenes; albeit unlikely given the low number of rRNA genes associated with mycoplasmas [78].

The presence of various bivalve-specific OTUs in this study also suggests that more explicit intrinsic (host) selection pressures may also be important in determining gut microbiota composition. Alongside the occurrence of various species-specific *Mycoplasmataceae*-related OTUs, for oysters this included OTUs representing the α-proteobacteria *Anaplasmataceae* (Rickettsiales) as well as *Spirochaetaceae* (Spirochaetota) and for mussels the *Spiroplasmataceae* (namely *Spiroplasma* sp.). Several of these organisms (notably *Anaplasmataceae*, *Mycoplasma* and Spirochaetota) have been reported to occur in association with infections arising from intracellular microcolonies of bacteria (IMC) in bivalves [79]. In some cases, IMC infections have the potential to cause widespread disease in certain farmed bivalve species [80, 81], though in earlier health surveys of *C. gigas* and *M. galloprovincialis*, the presence of specific IMC-related organisms (namely Rickettsia and *Mycoplasma*-like colonies) has not been associated with underlying pathology [82, 83]. Furthermore, in *M. galloprovincialis*, such organisms were found to increase in prevalence with growth during cultivation, where adults comprised the greatest proportion of these organisms compared to the seed [82]. Together, this supports earlier notions from other related bivalves of a perhaps more symbiotic relationship [84], which is likely established during host development. Given that the host-associated OTUs observed here were derived from samples from seemingly healthy individuals, such a relationship may appear more likely. Furthermore, with the occurrence of varied abundances of select host-associated OTUs between small and large mussels (notably those belonging to certain *Mycoplasma*-related taxa), it may appear that the relevance of these organisms may vary also with age and/or cohort specific genetics. Further insights into the presence and role of these taxa is thus warranted and may be expedited through in situ studies and/or phylogenomic-based investigations as conducted elsewhere [79, 85]. This may be of particular importance in order to exclude their occurrence as components introduced through potential dietary sources, as symbionts of microbial eukaryotes like protists [86].

In this study, the gut microbiota composition of oysters and mussels also appeared to be influenced by the month in which they were sampled. Most notable was an increase in species (OTU) richness and diversity in both oysters and mussels sampled in the late austral winter (Aug) and was associated with a variety of taxa, including several that exhibited a concomitant increase in prevalence in the bivalves and the corresponding seawater samples. In particular, during winter this included OTUs associated with largely heterotrophic taxa such as α-proteobacteria SAR11 clade 1a, *Rhodobacteraceae* (*Planktomarina* spp.), *Flavobacteriaceae* (NS5 marine group) and γ-proteobacteria SAR86 clade. As significant free-living or particle-associated constituents found in coastal and/or open-ocean waters throughout the world, and whose populations are well known to vary temporally [87–89], the occurrence of such taxa may reflect the common ingestion of local, environmentally driven microbial consortia, as discussed earlier. Indeed, bivalves in this study were sampled from a particularly dynamic region that, as part of the broader eastern Great Australian Bight (GAB), is marked by significant wind-driven summer upwelling and winter downwelling events that influence nutrient availability and mixing [90, 91]. During these periods, changes in the rates of productivity are observed and are the highest in the late summer upwelling season (typically January–April) when nutrient rich waters favour productivity and the lowest during winter downwelling conditions [91]. This is likely reflected here by the increased prevalence of particular cyanobacterial taxa in the bivalves during summer (e.g. *Synechococcus* spp.), who are associated with nutrient rich coastal waters [92] and have been found to be significant seasonal components of the environmental consortia that may be ingested by these bivalves in the region [58]. However, while an increase in their prevalence was not reflected in the seawater in summer, the occurrence of other OTUs such as *Methylophilaceae* (OM43 clade) which are associated with blooms [93, 94] may support their relevance here during these particular sampling months. Nevertheless, as discussed above, whether these organisms represent common (perhaps seasonally) ingested components of the diet or play a more direct role as part of the gut microbiota requires further examination. This may be particularly important for organisms like *Pseudoalteromonadaceae* (*Pseudoalteromonas* spp.) which also increased in abundance in bivalve and seawater samples during summer and which are associated with a variety of eukaryotic hosts (including oysters and mussels) [36, 95] and may confer a benefit through, e.g. antibiotic activity against pathogens [96].

Though extrinsic, seasonally relevant (environmental/particle diet) factors likely shape the gut microbiota of bivalves, as reported elsewhere [55, 97], the way in which they impact these communities also appears to vary between host species. Specifically, in this study, samples from oysters typically comprised a greater breadth of taxa in summer compared to winter, though were similarly evenly distributed across taxonomic lineages in both periods. In contrast, samples from mussels comprised a similar breadth of taxa in both summer and winter, though were more unevenly distributed across taxonomic lineages in winter compared to summer. Such disparity has also been previously reported to occur between other intergeneric bivalves held in the same environment, namely the eastern oyster (*C. virginica*) and blue mussel (*M. edulis*) [55]. However, unlike that observed here, greater evenness was observed for mussels rather than oysters across the seasons, though it was based on measurements of functional diversity (catabolic activity), which may fail to assess components of the community that require unusual substrates or are recalcitrant to cultivation [98*,* 99]. Nevertheless, at a holistic level, such differences may be reflective of the unique combinations of extrinsic and host-specific intrinsic factors that together shape the gut microbiota and which underpin their ecology and ability to share the same environment. This may be further exemplified by the apparent differences also observed at the individual level between bivalve samples and the summer and winter sampling months (most notably for oysters). Indeed, for oysters (*C. gigas*), it has been recently reported that high microbial evenness may confer enhanced resistance to infection by pertinent viral infections (namely ostreid herpesvirus 1, OsHV-1 μVar), possibly through the promotion of homeostasis [28]. The prevalence of select taxa in the individual bivalves during the summer or winter months, however, may also confer specific benefits. For oysters, this may be reflected in the increased prevalence of certain taxa like *Pseudomonas pseudoalcaligenes* in winter, which known for its capacity to accumulate or breakdown harmful compounds (e.g., mercury, polychlorinated biphenyls) [100, 101] and may help to overcome potential stressors posed by such compounds for growth and reproduction [102]. Conversely, for mussels, a greater proportion of Spirochaetota in summer, as a saccharolytic organism thought to mediate the turnover of algal detritus [103], may support nutrition by breaking down the cellulolytic components of the diet, as observed in other mytilids [104].

The majority of OTUs contributing to the observed differences between the summer and winter sampling months in both oysters and mussels, however, included those most closely related to members of the *Mycoplasmataceae*, whereby each comprised a number of distinct OTUs that were either more prevalent in summer or winter. Given that such organisms are not typically considered free-living, but instead are reliant upon their host for supporting their metabolic requirements [70], such changes were somewhat unexpected. Interestingly, alongside their reported absence from suspended marine aggregates (particle diets), seasonal changes in the abundance of the Mycoplasmatales have also been observed previously for oysters (*C. virginica*) and mussels (*M. edulis*) [55]. While it is not possible to exclude other factors that may influence the prevalence of these organisms (e.g. environmental conditions or competition with other microbes), such a finding raises some intriguing questions. Specifically, whether the host may be directly controlling these populations in order to support seasonal changes in its physiology or metabolic requirements, a feature observed for lucinid bivalves where its symbionts are enzymatically digested during starvation [105]. Further, more detailed investigations over a greater period (encompassing multiple time points across all seasons) and using RNA instead of DNA to discern the active and thus likely resident constituents of the microbiota are thus needed in order to better determine the association and dynamics these organisms share with the bivalve host. In addition, given the approach used in this study likely considers the more predominant taxa (where OTUs with an accumulative abundance of < 0.01% were filtered from the final dataset), further work should also be directed to explore the ‘rare’ constituents of the bivalve gut bacterial community.

## Conclusions

This study has shown that both a large common (core) microbiota and bivalve-specific host-associated and environmental (free-living or particle-diet associated) taxa may occur within intergeneric, cohabitating bivalves, including taxa that have become increasingly realised as important constituents of the gut microbiota of other marine animals such as the Mollicutes (*Mycoplasmataceae*). Changes in the prevalence of these taxa between summer and winter point towards possible varied strategies employed by the host (likely as part of its feeding ecology) at different times of the year and raise intriguing questions around the level of control the host exerts in selecting/regulating its gut assemblages, particularly in the case of potentially parasitic/symbiotic taxa like *Mycoplasma*.

## Supplementary Information

ESM 1: Supplementary Datasheet 1 Relative abundances of OTUs and their mean abundances, taxonomic assignment, representative sequences and identity scores from seawater and gut aspirate samples obtained from mussels and oysters in this study. (XLSX 768 kb)

ESM 2: **Supplementary Table 1** List of the significant differentially abundant phyla observed from oyster and mussel gut samples obtained in summer and winter as determined using the Kruskal-Wallis rank test (adjusted p-value [FDR] cut-off = 0.01). The Log LDA Score cut-off value was adjusted to 1.0 and significant taxa are given in descending order from the highest to lowest LDA score. Mean abundances (%) are presented for each, with ranges given in parentheses. **Supplementary Table 2** List of the unique OTUs detected from oyster gut samples following Illumina-based 16S rRNA (V1-V2) gene sequencing, including their classification, percent identity and closest sequence in RDP. Highlighted OTUs were not detected from seawater, and the top three most abundant (irrespective of the summer or winter month of sampling) are given in bold. **Supplementary Table 3** List of the unique OTUs detected from oyster gut samples following Illumina-based 16S rRNA (V1-V2) gene sequencing, including their classification, percent identity and closest sequence in RDP. Highlighted OTUs were not detected from seawater, and the top three most abundant (irrespective of the summer or winter month of sampling) are given in bold. **Supplementary Table 4** List of the significant differentially abundant OTUs observed from large and small summer mussel samples as determined using the Kruskal-Wallis rank test (unadjusted *p*-value cut off = 0.01). The Log LDA Score cut-off value was adjusted to 1.0 and significant taxa are given in descending order from the highest to lowest LDA score. Mean abundances (%) are presented for each, with ranges given in parentheses. **Supplementary Table 5** List of the top 20 significant differentially abundant families observed from oyster and mussel gut and seawater samples obtained in summer and winter as determined using the Kruskal-Wallis rank test (adjusted p-value [FDR] cut-off = 0.01). The Log LDA Score cut-off value was adjusted to 2.0 and significant taxa are given in descending order from the highest to lowest LDA score. Mean abundances (%) are presented for each, with ranges given in parentheses. **Supplementary Table 6** List of the top 20 significant differentially abundant OTUs observed from oyster and mussel gut and seawater samples obtained in summer and winter as determined using the Kruskal-Wallis rank test (adjusted p-value [FDR] cut-off = 0.01). The Log LDA Score cut-off value was adjusted to 2.0 and significant taxa are given in descending order from the highest to lowest LDA score. Mean abundances (%) are presented for each, with ranges given in parentheses. (DOCX 3.83 MB)

## Data Availability

The OTU abundance table used for the associated analyses is provided in Supplementary Datasheet 1. Sequences from individual samples were deposited within the NCBI under Bioproject ID PRJNA845084 (accession numbers SAMN23375497–SAMN23375618).
