# Supplementary material for: Host Species and Environment Shape the Gut Microbiota of Cohabiting Marine Bivalves
Source: Microb Ecol. 2023 Feb 22;86(3):1755–72. doi: 10.1007/s00248-023-02192-z (PMC10497454; doi:10.1007/s00248-023-02192-z)
Supplement: Supplementary file 2 — Supplementary Table 1 List of the significant differentially abundant phyla observed from oyster and mussel gut samples obtained in summer and winter as determined using the Kruskal-Wallis rank test (adjusted p-value [FDR] cut-off = 0.01). The Log LDA Score cut-off value was adjusted to 1.0 and significant taxa are given in descending order from the highest to lowest LDA score. Mean abundances (%) are presented for each, with ranges given in parentheses. Supplementary Table 2 List of the unique OTUs detected from oyster gut samples following Illumina-based 16S rRNA (V1-V2) gene sequencing, including their classification, percent identity and closest sequence in RDP. Highlighted OTUs were not detected from seawater, and the top three most abundant (irrespective of the summer or winter month of sampling) are given in bold. Supplementary Table 3 List of the unique OTUs detected from oyster gut samples following Illumina-based 16S rRNA (V1-V2) gene sequencing, including their classification, percent identity and closest sequence in RDP. Highlighted OTUs were not detected from seawater, and the top three most abundant (irrespective of the summer or winter month of sampling) are given in bold. Supplementary Table 4 List of the significant differentially abundant OTUs observed from large and small summer mussel samples as determined using the Kruskal-Wallis rank test (unadjusted p-value cut off = 0.01). The Log LDA Score cut-off value was adjusted to 1.0 and significant taxa are given in descending order from the highest to lowest LDA score. Mean abundances (%) are presented for each, with ranges given in parentheses. Supplementary Table 5 List of the top 20 significant differentially abundant families observed from oyster and mussel gut and seawater samples obtained in summer and winter as determined using the Kruskal-Wallis rank test (adjusted p-value [FDR] cut-off = 0.01). The Log LDA Score cut-off value was adjusted to 2.0 and significant taxa are given in descending o [file 248_2023_2192_MOESM2_ESM.docx]

**Supplementary information for:**

**Host species and environment shape the gut microbiota of cohabiting marine bivalves**

Shirin Akter^1^, Melissa L Wos-Oxley^2^, Sarah R Catalano^3^, Md Mahbubul Hassan^1,4^, Xiaoxu Li^3^, Jian G Qin^1^, Andrew PA Oxley^5*^

^1^College of Science and Engineering, Flinders University, Adelaide, SA, Australia.

^2^School of Medicine, Deakin University, Geelong, VIC, Australia

^3^Aquatic Sciences Centre, South Australian Research and Development Institute, West Beach, SA, Australia

^4^Aquaculture Research and Development, Department of Primary Industries and Regional Development, Hillarys, WA, Australia

^5^School of Life and Environmental Sciences, Deakin University, Geelong, VIC, Australia

***Corresponding author:**

**Andrew PA Oxley**: Deakin University, School of Life and Environmental Sciences,

Geelong Waurn Ponds Campus, 75 Pigdons Road,

Waurn Ponds, VIC 3216, Australia. Tel.: +61 3 522 73670; Email: [Andrew.Oxley@deakin.edu.au](mailto:Andrew.Oxley@deakin.edu.au)

**
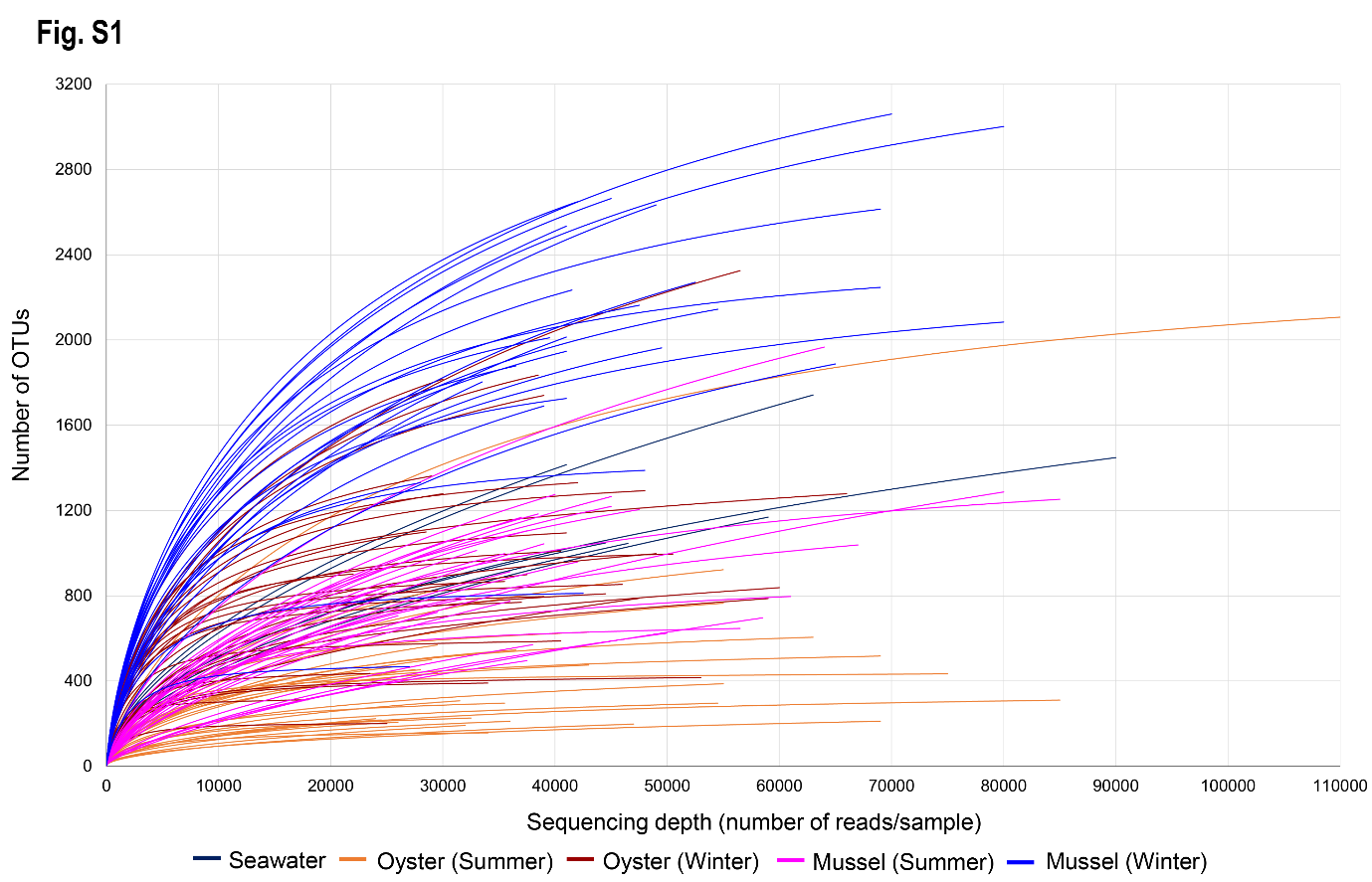
**

**Supplementary Fig. 1** Rarefaction curves depicting the number of resolved OTUs against sequencing depth of the 122 samples collected in summer and winter (6 seawater, and 60 oyster and 56 mussel gut samples).


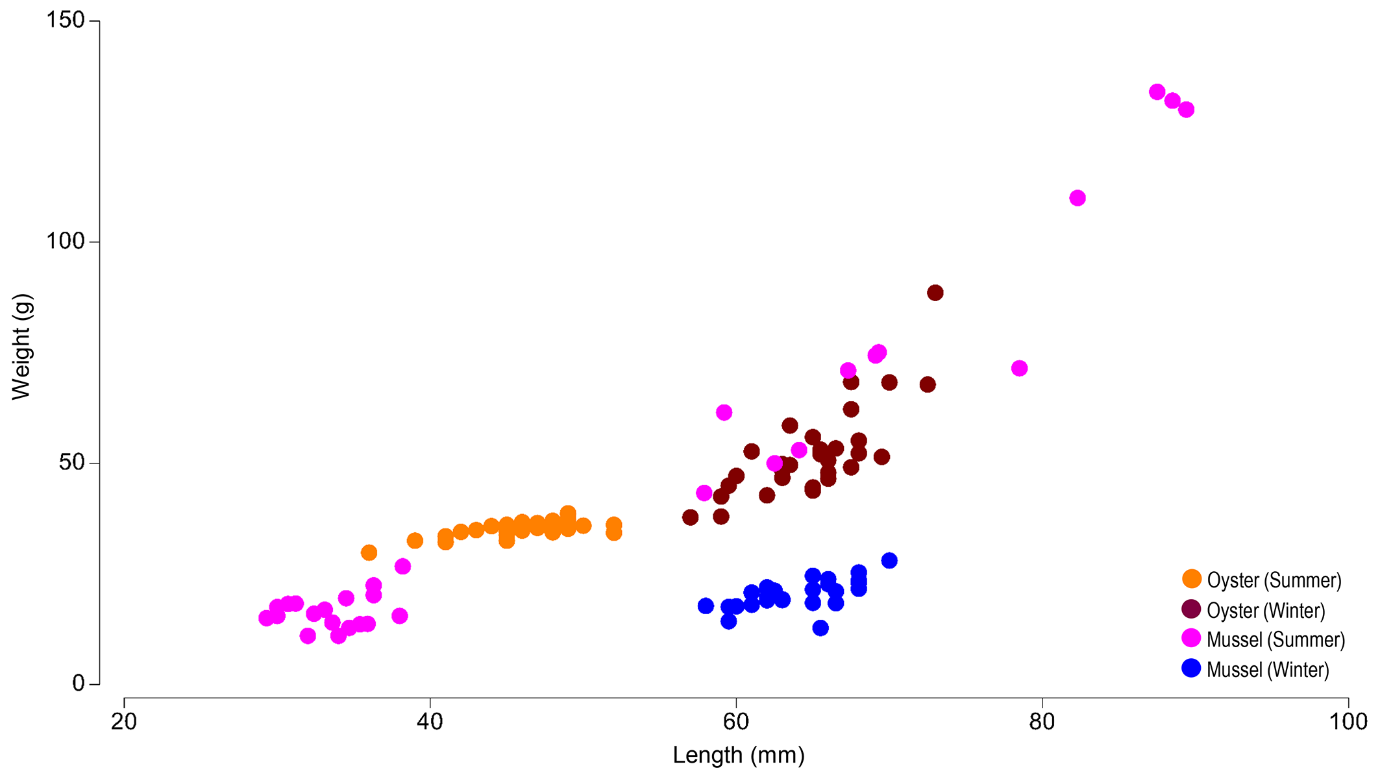


**Supplementary Fig. 2** Scatter plot displaying the lengths vs weights of oyster and mussels collected in summer and winter for bacterial community analysis from Coffin Bay, South Australia.


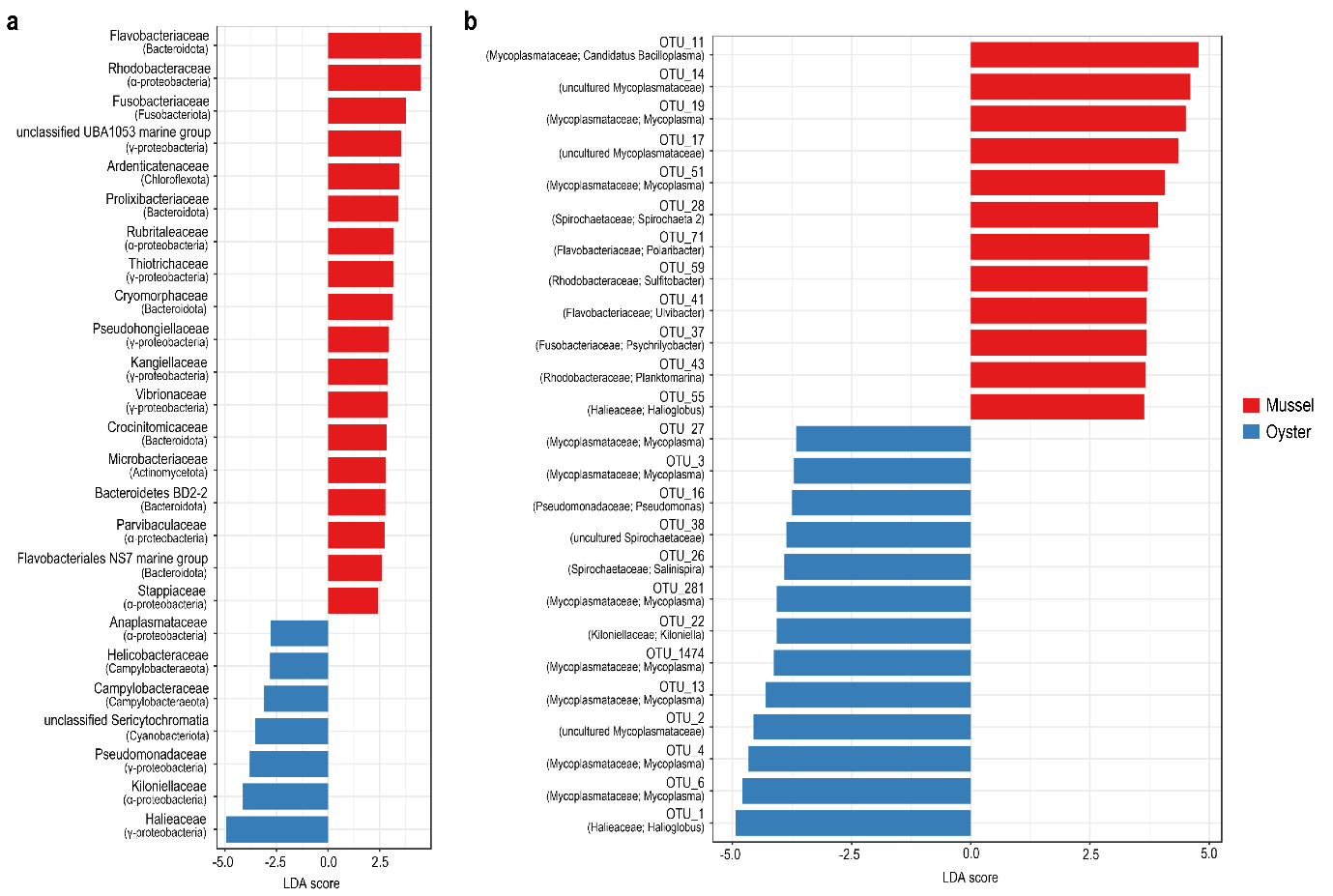


**Supplementary Fig. 3** Linear Discriminant Analysis (LDA) Effect Size (LEfSe) bar plots displaying the differentially abundant bacterial families (A) and top 25 OTUs (B) from mussel and oyster gut samples obtained in from Coffin Bay, South Australia (irrespective of the summer or winter month of sampling). Differentially abundant features were determined using the Kruskal-Wallis rank test (adjusted *p*-value [FDR] cut-off = 0.01), with the Log LDA Score value adjusted to 2.0 and significant taxa given in descending order from the highest to lowest LDA score.

| **Phyla** | **FDR** | **LDA score** | **Mean abundance (%)** | | | |
| --- | --- | --- | --- | --- | --- | --- |
|  |  |  | **Mussels (summer)** | **Mussels (winter)** | **Oysters (summer)** | **Oysters (winter)** |
| Mycoplasmatota | 1.32E-13 | 5.44 | 80.806 (min 56.999, max 92.052) | 36.112 (min 8.0639, max 74.185) | 64.148 (min 23.744, max 91.952) | 26.794 (min 3.169, max 78.608) |
| Pseudomonadota | 5.31E-15 | 5.4 | 6.252 (min 2.782, max 13.796) | 35.989 (min 16.201, max 56.143) | 21.319 (min 5.176, max 57.648) | 56.697 (min 14.913, max 87.219) |
| Bacteroidota | 1.59E-15 | 4.82 | 1.622 (min 0.481, max 5.766) | 14.030 (min 2.190, max 23.387) | 0.502 (min 0.042, max 4.023) | 1.749 (min 0.430, max 5.536) |
| Spirochaetota | 3.03E-11 | 4.46 | 3.363 (min 0.320, max 14.052) | 0.537 (min 0, max 2.938) | 6.485 (min 0.417, max 37.876) | 0.669 (min 0, max 4.613) |
| Actinomycetota | 4.52E-15 | 4.38 | 0.398 (min 0.119, max 1.121) | 4.511 (min 1.397, max 11.433) | 0.542 (min 0.023, max 2.260) | 5.208 (min 0.938, max 14.344) |
| Fusobacteriota | 1.94E-08 | 4 | 2.134 (min 0.185, max 8.864) | 0.735 (min 0, max 8.335) | 0.113 (min 0, max 0.803) | 0.141 (min 0, max 1.486) |
| Bacillota | 4.52E-15 | 3.83 | 0.100 (min 0.020, max 0.267) | 1.049 (min 0.429, max 2.626) | 0.178 (min 0.006, max 0.616) | 1.448 (min 0.2956, max 2.901) |
| Chloroflexota | 1.89E-15 | 3.77 | 0.016 (min 0.002, max 0.050) | 1.186 (min 0.072, max 3.906) | 0.016 (min 0, max 0.075) | 0.334 (min 0, max 1.010) |
| Campylobacteraeota | 5.18E-11 | 3.5 | 0.010 (min 0, max 0.026) | 0.258 (min 0.110, max 0.449) | 0.413 (min 0 max 1.248) | 0.647 (min 0.136 max 2.424) |
| Verrucomicrobiota | 2.18E-13 | 3.4 | 0.026 (min 0, max 0.090) | 0.507 (min 0.005, max 1.375) | 0.013 (min 0, max 0.091) | 0.009 (min 0, max 0.041) |
| Acidobacteriota | 6.60E-11 | 2.91 | 0.004 (min 0, max 0.013) | 0.090 (min 0, max 0.264) | 0.004 (min 0, max 0.030) | 0.165 (min 0, max 0.468) |

**Supplementary Table 1** List of the significant differentially abundant phyla observed from oyster and mussel gut samples obtained in summer and winter as determined using the Kruskal-Wallis rank test (adjusted p-value [FDR] cut-off = 0.01). The Log LDA Score cut-off value was adjusted to 1.0 and significant taxa are given in descending order from the highest to lowest LDA score. Mean abundances (%) are presented for each, with ranges given in parentheses.

| **OTU #** | **Mean abundance (%)** | | | |  | |
| --- | --- | --- | --- | --- | --- | --- |
|  | **Oysters Summer** | **Oysters Winter** | **Seawater Summer** | **Seawater Winter** | **Identity (%)** | **Classification** |
| OTU_224 | 0.04811 | 0.02777 | 0 | 0 | 70.43 | Pseudomonadota; Gammaproteobacteria; unclassified Gammaproteobacteria; uncultured bacterium_JF827373 |
| OTU_14707 | 0.03617 | 0.00321 | 0 | 0 | 80.33 | Mycoplasmatota; Mollicutes; Mycoplasmatales; Mycoplasmataceae; Mycoplasma; uncultured bacterium_GU070687 |
| OTU_140 | 0.00991 | **0.22122** | 0 | 0 | 69.39 | Pseudomonadota; Alphaproteobacteria; Rickettsiales; Anaplasmataceae; Candidatus Neoehrlichia; Candidatus Thiopilula aggregata_FR690980 |
| OTU_553 | 0.00018 | 0.00248 | 0.14208 | 0.08345 | 100.00 | Actinomycetota; Acidimicrobiia; Actinomarinales; Actinomarinaceae; Candidatus Actinomarina; uncultured actinobacterium MB11A03_AY033296 |
| OTU_1570 | 0.00016 | 0.00043 | 0.00042 | 0 | 90.50 | Ca. Latescibacteria; Latescibacteria; Latescibacterales; Latescibacteraceae; unclassified Latescibacteraceae; uncultured bacterium_JN092240 |
| OTU_20272 | 0.00010 | 0.00243 | 0.02589 | 0.03117 | 96.92 | Bacteroidota; Bacteroidia; Flavobacteriales; Cryomorphaceae; uncultured Cryomorphaceae; uncultured marine bacterium_HM437434 |
| OTU_263 | **0.11918** | 0 | 0 | 0 | 82.23 | Spirochaetota; Spirochaetia; Spirochaetales; Spirochaetaceae; Spirochaeta 2; uncultured bacterium_JF827378 |
| OTU_9073 | **0.09950** | 0 | 0 | 0 | 80.33 | Mycoplasmatota; Mollicutes; Mycoplasmatales; Mycoplasmataceae; Mycoplasma; uncultured bacterium_FM995178 |
| OTU_4363 | 0.05387 | 0 | 0 | 0 | 79.59 | Mycoplasmatota; Mollicutes; Mycoplasmatales; Mycoplasmataceae; Mycoplasma; uncultured bacterium_JF827476 |
| OTU_77 | 0.02312 | 0 | 0 | 0 | 71.49 | Bacillota; Erysipelotrichia; Erysipelotrichales; Erysipelotrichaceae; Dielma; uncultured bacterium_FN550075 |
| OTU_276 | 0.00048 | 0 | 0 | 0 | 84.58 | Cyanobacteriota; Oxyphotobacteria; Nostocales; Chroococcidiopsaceae; uncultured cyanobacterium_JQ402424 |
| OTU_17632 | 0.00047 | 0 | 0 | 0.08531 | 98.75 | Pseudomonadota; Gammaproteobacteria; Oceanospirillales; Saccharospirillaceae; Oleispira; uncultured gamma proteobacterium_AF353238 |
| OTU_759 | 0.00041 | 0 | 0.46333 | 0 | 98.75 | Pseudomonadota; Gammaproteobacteria; Alteromonadales; Alteromonadaceae; Alteromonas; HMWdDNA-degrading marine bacterium 31_EF103087 |
| OTU_681 | 0.00030 | 0 | 0.36095 | 0 | 87.50 | Bacteroidota; Bacteroidia; Flavobacteriales; Crocinitomicaceae; Crocinitomix; uncultured bacterium_JQ214458 |
| OTU_550 | 0.00027 | 0 | 0.43627 | 0 | 92.92 | Pseudomonadota; Gammaproteobacteria; Alteromonadales; Alteromonadaceae; Glaciecola; uncultured gamma proteobacterium CHAB-XII-8_AJ240915 |
| OTU_638 | 0.00023 | 0 | 0 | 0 | 90.87 | Pseudomonadota; Gammaproteobacteria; Cellvibrionales; Cellvibrionaceae; Marinagarivorans; uncultured Alteromonadales bacterium_AY712105 |
| OTU_858 | 0.00018 | 0 | 0.08550 | 0 | 97.92 | Bacteroidota; Bacteroidia; Chitinophagales; Saprospiraceae; Aureispira; uncultured bacterium_EF573760 |
| OTU_2948 | 0.00014 | 0 | 0 | 0 | 96.28 | Bacteroidota; Bacteroidia; Flavobacteriales; Flavobacteriaceae; Polaribacter 3; uncultured marine bacterium_FJ826130 |
| OTU_893 | 0.00013 | 0 | 0.15291 | 0 | 88.84 | Bacteroidota; Bacteroidia; Flavobacteriales; Crocinitomicaceae; Fluviicola; uncultured Flavobacterium sp._JN233129 |
| OTU_1081 | 0.00011 | 0 | 0 | 0.11076 | 85.48 | Bacteroidota; Bacteroidia; Flavobacteriales; Cryomorphaceae; uncultured Flavobacterium sp._FJ745267 |
| OTU_546 | 0 | 0.00340 | 0.01799 | 0.13008 | 95.83 | Bacteroidota; Bacteroidia; Flavobacteriales; unclassified Flavobacteriales NS9 marine group; uncultured marine bacterium_FN435390 |
| OTU_2449 | 0 | 0.00215 | 0.00166 | 0.02087 | 100.00 | Pseudomonadota; Gammaproteobacteria; unclassified Gammaproteobacteria SAR86 clade; uncultured bacterium_AF382123 |
| OTU_6362 | 0 | 0.00155 | 0.00653 | 0.00653 | 89.58 | Bacteroidota; Bacteroidia; Flavobacteriales; Flavobacteriaceae; Flavobacteriaceae NS5 marine group; uncultured bacterium_EU636597 |
| OTU_2099 | 0 | 0.00061 | 0 | 0 | 92.89 | Pseudomonadota; Gammaproteobacteria; Alteromonadales; Colwelliaceae; Thalassotalea; uncultured bacterium_HF558550 |
| OTU_1939 | 0 | 0.00030 | 0.00176 | 0.01271 | 93.78 | Pseudomonadota; Gammaproteobacteria; Thiotrichales; Thiotrichaceae; uncultured bacterium_JX864308 |
| OTU_1945 | 0 | 0.00027 | 0 | 0.06934 | 95.85 | Bacteroidota; Bacteroidia; Flavobacteriales; unclassified Flavobacteriales NS7 marine group; uncultured marine bacterium_GU235567 |
| OTU_3559 | 0 | 0.00012 | 0 | 0.03481 | 97.07 | Pseudomonadota; Alphaproteobacteria; unclassified Alphaproteobacteria SAR11 clade I; uncultured bacterium_JQ195102 |
| OTU_2656 | 0 | 0.00011 | 0 | 0 | 85.54 | Bacteroidota; Bacteroidia; Flavobacteriales; Flavobacteriaceae; Psychroflexus; uncultured Bacteroidetes bacterium_FJ213812 |

**Supplementary Table 2** List of the unique OTUs detected from oyster gut samples following Illumina-based 16S rRNA (V1-V2) gene sequencing, including their classification, percent identity and closest sequence in RDP. Highlighted OTUs were not detected from seawater, and the top three most abundant (irrespective of the summer or winter month of sampling) are given in bold.

| **OTU #** | **Mean abundance (%)** | | | |  |  |
| --- | --- | --- | --- | --- | --- | --- |
|  | **Mussels Summer** | **Mussels Winter** | **Seawater Summer** | **Seawater Winter** | **Identity (%)** | **Classification** |
| OTU_115 | **0.52595** | 0.02939 | 0 | 0 | 71.95 | Mycoplasmatota; Mollicutes; Mycoplasmatales; Mycoplasmataceae; Mycoplasma; Mycoplasma genitalium M6320_CP003772 |
| OTU_261 | **0.17323** | 0.04078 | 0 | 0 | 82.11 | Mycoplasmatota; Mollicutes; Mycoplasmatales; Mycoplasmataceae; Mycoplasma; uncultured bacterium_FM995178 |
| OTU_677 | 0.00035 | 0 | 0 | 0 | 89.63 | Bacteroidota; Bacteroidia; Flavobacteriales; Flavobacteriaceae; Bizionia; Bizionia psychrotolerans T_KJ461691 |
| OTU_1571 | 0 | 0.00176 | 0 | 0 | 94.17 | Campylobacteraeota; Campylobacteria; Campylobacterales; Arcobacteraceae; Arcobacter; uncultured epsilon proteobacterium_HE576785 |
| OTU_2403 | 0 | 0.00102 | 0 | 0 | 90.72 | Bacteroidota; Bacteroidia; Flavobacteriales; Cryomorphaceae; uncultured Bacteroidetes bacterium_GQ274047 |
| OTU_1448 | 0 | 0.00072 | 0 | 0 | 78.84 | Pseudomonadota; Alphaproteobacteria; Rhodospirillales; uncultured bacterium_KC465655 |
| OTU_1396 | 0 | 0.00013 | 0 | 0 | 87.55 | Bacteroidota; Bacteroidia; Flavobacteriales; Crocinitomicaceae; uncultured bacterium_EU045077 |
| OTU_180 | **0.22770** | 0 | 0 | 0.00089 | 71.37 | Mycoplasmatota; Mollicutes; Entomoplasmatales; Spiroplasmataceae; Spiroplasma; Mycoplasma fastidiosum T_AF125878 |
| OTU_1982 | 0.00116 | 0.00315 | 0.00297 | 0 | 97.50 | Bacteroidota; Bacteroidia; Flavobacteriales; Flavobacteriaceae; Kordia; Cytophaga sp._AB017046 |
| OTU_2113 | 0.00100 | 0.00731 | 0.00340 | 0 | 92.50 | Bacteroidota; Bacteroidia; Flavobacteriales; Flavobacteriaceae; Flavobacterium; uncultured bacterium_JF827501 |
| OTU_68 | 0.00099 | 0.00010 | 0.01507 | 0.00060 | 96.25 | Bacteroidota; Bacteroidia; Flavobacteriales; Flavobacteriaceae; Polaribacter 4; uncultured Flavobacteriales bacterium_KF786569 |
| OTU_385 | 0.00087 | 0 | 0 | 0.00089 | 92.56 | Pseudomonadota; Gammaproteobacteria; Cellvibrionales; Spongiibacteraceae BD1-7 clade; uncultured bacterium_JX015952 |
| OTU_2787 | 0.00051 | 0.00126 | 0 | 0.01268 | 88.02 | Bacteroidota; Bacteroidia; Flavobacteriales; Flavobacteriaceae NS5 marine group; uncultured bacterium_EF379585 |
| OTU_1476 | 0.00016 | 0.00019 | 0 | 0.00353 | 87.97 | Pseudomonadota; Gammaproteobacteria; Oceanospirillales; Saccharospirillaceae; Thalassolituus; uncultured Marinobacter sp._HM216500 |
| OTU_7972 | 0.00014 | 0 | 0.07826 | 0.00534 | 98.33 | Pseudomonadota; Alphaproteobacteria; Alphaproteobacteria SAR11 clade II; uncultured bacterium_FJ744894 |
| OTU_2115 | 0.00012 | 0.00013 | 0.01502 | 0.01309 | 99.58 | Pseudomonadota; Alphaproteobacteria; Rhodospirillales AEGEAN-169 marine group; uncultured bacterium_GU461167 |
| OTU_2349 | 0.00010 | 0 | 0.04043 | 0.00551 | 85.48 | Bacteroidota; Bacteroidia; Chitinophagales; Saprospiraceae; Lewinella; uncultured Bacteroidetes bacterium_GQ274081 |
| OTU_310 | 0.00010 | 0 | 0.00848 | 0 | 88.29 | Fusobacteriota; Fusobacteriia; Fusobacteriales; Leptotrichiaceae; Hypnocyclicus; uncultured Fusobacteria bacterium_DQ206413 |
| OTU_5711 | 0.00006 | 0.00182 | 0.00085 | 0.00178 | 96.67 | Pseudomonadota; Gammaproteobacteria; Alteromonadales; Colwelliaceae; Colwellia; Alteromonadaceae bacterium SN-1009_AB097610 |
| OTU_1515 | 0 | 0.00241 | 0.00574 | 0.00114 | 85.95 | Pseudomonadota; Alphaproteobacteria; Rhodospirillales; Terasakiellaceae; alpha proteobacterium 'Egg clone D38'_AF022399 |
| OTU_1315 | 0 | 0.00217 | 0.00258 | 0.01085 | 87.14 | Bacteroidota; Bacteroidia; Chitinophagales; uncultured Bacteroidetes bacterium_GQ347704 |
| OTU_3010 | 0 | 0.00072 | 0 | 0.04005 | 91.67 | Pseudomonadota; Alphaproteobacteria; Puniceispirillales SAR116 clade; uncultured bacterium_JQ196540 |
| OTU_778 | 0 | 0.00057 | 0 | 0.00260 | 95.42 | Pseudomonadota; Gammaproteobacteria; Alteromonadales; Colwelliaceae; Thalassotalea; Thalassotalea piscium T_JX412957 |
| OTU_2240 | 0 | 0.00040 | 0.00131 | 0.01342 | 97.50 | Pseudomonadota; Alphaproteobacteria; Thalassobaculales; Nisaeaceae OM75 clade; uncultured bacterium_EU799269 |
| OTU_1675 | 0 | 0.00027 | 0 | 0.00178 | 90.83 | Pseudomonadota; Gammaproteobacteria; Alteromonadales; Colwelliaceae; Colwellia; Colwellia asteriadis T_EU599214 |
| OTU_3329 | 0 | 0.00022 | 0 | 0.04210 | 99.17 | Pseudomonadota; Alphaproteobacteria; Alphaproteobacteria SAR11 clade II; uncultured bacterium_JF509025 |
| OTU_2619 | 0 | 0.00016 | 0 | 0.05775 | 97.08 | Pseudomonadota; Alphaproteobacteria; Alphaproteobacteria SAR11 clade Ib; uncultured marine bacterium_DQ071161 |
| OTU_1389 | 0 | 0.00007 | 0.06859 | 0.00089 | 97.48 | Pseudomonadota; Alphaproteobacteria; Parvibaculales; Parvibaculaceae; uncultured bacterium_JN018677 |

**Supplementary Table 3** List of the unique OTUs detected from oyster gut samples following Illumina-based 16S rRNA (V1-V2) gene sequencing, including their classification, percent identity and closest sequence in RDP. Highlighted OTUs were not detected from seawater, and the top three most abundant (irrespective of the summer or winter month of sampling) are given in bold.

| **OTU #** | **Classification** | ***p-value*** | **LDA score** | **Mean abundance (%)** | |
| --- | --- | --- | --- | --- | --- |
|  |  |  |  | **Small mussels (<40mm)** | **Large mussels (>60mm)** |
| OTU_51 | Mycoplasmatota; Mycoplasmataceae, Mycoplasma | 0.00037654 | -4.99 | 3.770 (min 0.1511, max 15.604) | 23.496 (min 1.493, max 86.585) |
| OTU_115 | Mycoplasmatota; Mycoplasmataceae, Mycoplasma | 0.0013985 | 3.48 | 0.769 (min 0.003, max 3.598) | 0.161 (min 0, max 1.814) |
| OTU_79 | Bacteroidota; Cyclobacteriaceae; Marinoscillum | 0.0042272 | 1.88 | 0.021 (min 0, max 0.083) | 0.006 (min 0, max 0.017) |
| OTU_44 | Bacteroidota; Flavobacteriaceae NS5 marine group | 0.0047105 | 2.1 | 0.035 (min 0, max 0.331) | 0.010  (min 0, max 0.068) |
| OTU_167 | Mycoplasmatota; Mycoplasmataceae, Mycoplasma | 0.0079834 | 2.81 | 0.135 (min 0, max 1.374) | 0.008 (min 0, max 0.089) |

**Supplementary Table 4**  List of the significant differentially abundant OTUs observed from large and small summer mussel samples as determined using the Kruskal-Wallis rank test (unadjusted *p*-value cut off = 0.01). The Log LDA Score cut-off value was adjusted to 1.0 and significant taxa are given in descending order from the highest to lowest LDA score. Mean abundances (%) are presented for each, with ranges given in parentheses.

| **Classification** | **FDR** | **LDA score** | **Mean abundance (%)** | | | | | |
| --- | --- | --- | --- | --- | --- | --- | --- | --- |
|  |  |  | **Mussels (summer)** | **Mussels (winter)** | **Oysters (summer)** | **Oysters (winter)** | **Seawater (summer)** | **Seawater (winter)** |
| Mycoplasmatota; Mycoplasmataceae | 5.885E-14 | 5.6 | 80.393 (min 56.989, max 90.718) | 36.112 (min 8.063, max 74.185) | 64.148 (min 23.744, max 91.952) | 26.794 (min 3.169, max 78.608) | 0.064 (min 0.009, max 0.119) | 0.097 (min 0.013, max 0.219) |
| α-proteobacteria; unclassified SAR11 clade Ia | 1.117E-08 | 5.29 | 0.011 (min 0, max 0.041) | 0.187 (min 0.031, max 0.718) | 0.090 (min 0, max 0.654) | 0.863 (min 0, max 15.962) | 36.940 (min 35.795, max 38.789) | 40.437 (min 39.460, max 41.358) |
| γ-proteobacteria; Halieaceae | 4.416E-15 | 5.03 | 0.853 (min 0.104, max 3.869) | 2.729 (min 0.855, max 8.922) | 15.906 (min 2.147, max 44.868) | 21.729 (min 2.875, max 70.280) | 0.419 (min 0.306, max 0.563) | 0.522 (min 0.361, max 0.607) |
| α-proteobacteria; Rhodobacteraceae | 1.003E-14 | 4.92 | 1.555 (min 0.3674, max 3.984) | 17.573 (min 5.459, max 33.264) | 0.966 (min 0.038, max 3.961) | 9.139 (min 0.527, max 25.871) | 9.955 (min 8.341, max 12.402) | 13.051 (min 6.763, max 17.741) |
| Bacteroidota; Flavobacteriaceae | 6.365E-16 | 4.7 | 0.744 (min 0.130, max 2.103) | 11.596 (min 1.219, max 20.752) | 0.216 (min 0.009, max 0.978) | 1.145 (min 0.090, max 3.955) | 7.987 (min 7.091, max 8.982) | 11.070 (min 10.415, max 12.099) |
| Spirochaetota; Spirochaetaceae | 6.457E-12 | 4.5 | 3.363 (min 0.320, max 14.052) | 0.537 (min 0, max 2.938) | 6.485 (min 0.417, max 37.876) | 0.669 (min 0, max 4.613) | 0.002 (min 0, max 0.005) | 0.003 (min 0, max 0.005) |
| α-proteobacteria; Kiloniellaceae | 4.850E-07 | 4.45 | 1.464 (min 0.017, max 8.317) | 0.272 (min 0.055, max 0.475) | 1.335 (min 0, max 14.738) | 5.627 (min 0.086, max 48.963) | 0.001 (min 0, max 0.002) | 0.002 (min 0, max 0.005) |
| γ-proteobacteria; Burkholderiaceae | 2.626E-14 | 4.44 | 0.106 (min 0.017, max 0.405) | 2.427 (min 0.163, max 14.533) | 0.509 (min 0.084, max 3.882) | 5.573 (min 0.192, max 37.924) | 0.004 (min 0, max 0.009) | 0.009 (min 0.004, max 0.016) |
| Cyanobacteriota; Cyanobiaceae | 8.921E-03 | 4.36 | 5.034 (min 1.554, max 14.152) | 3.715 (min 1.612, max 7.984) | 5.627 (min 0.080, max 20.157) | 3.482 (min 0.223, max 8.507) | 1.396 (min 1.237, max 1.522) | 1.284 (min 1.087, max 1.640) |
| γ-proteobacteria; Pseudomonadaceae | 1.322E-12 | 4.32 | 0.051 (min 0.003, max 0.229) | 2.066 (min 0.093, max 26.018) | 0.685 (min 0.027, max 4.273) | 4.195 (min 0.125, max 22.467) | 0.007 (min 0.003, max 0.013) | 0.006 (min 0.004, max 0.008) |
| α-proteobacteria; Rhizobiaceae | 1.901E-15 | 4.19 | 0.197 (min 0.031, max 0.454) | 3.074 (min 0.248, max 5.923) | 0.115 (min 0.022, max 0.573) | 1.863 (min 0.197, max 5.016) | 0.008 (min 0.007, max 0.010) | 0.035 (min 0.022, max 0.048) |
| Bacteroidota; Cryomorphaceae | 8.280E-13 | 4.13 | 0.191 (min 0.045, max 0.569) | 0.564 (min 0.055, max 1.465) | 0.037 (min 0, max 0.216) | 0.188 (min 0, max 1.029) | 6.758 (min 6.056, max 7.752) | 3.614 (min 3.044, max 4.121) |
| Actinomycetota; Microbacteriaceae | 1.070E-11 | 4.11 | 0.001 (min 0, max 0.013) | 0.248 (min 0, max 1.694) | 0.006 (min 0, max 0.045) | 0.048 (min 0, max 1.180) | 2.967 (min 2.614, max 3.201) | 6.397 (min 5.672, max 6.809) |
| δ-proteobacteria; Desulfobulbaceae | 4.550E-15 | 4.08 | 0.104 (min 0.037, max 0.285) | 1.245 (min 0.351, max 3.675) | 0.107 (min 0, max 0.568) | 2.395 (min 0.147, max 7.137) | 0.013 (min 0.009, max 0.018) | 0.062 (min 0.042, max 0.073) |
| Cyanobacteriota; unclassified Sericytochromatia | 3.787E-12 | 4.06 | 0.152 (min 0.029, max 1.049) | 1.162 (min 0.141, max 7.917) | 0.545 (min 0.152, max 1.899) | 2.290 (min 0.102, max 12.470) | 0.004 (min 0.002, max 0.005) | 0.008 (min 0.005, max 0.011) |
| γ-proteobacteria; unclassified SAR86 clade | 1.472E-08 | 4.06 | 0.010 (min 0, max 0.093) | 0.054 (min 0, max 0.263) | 0.007 (min 0, max 0.062) | 0.084 (min 0, max 1.796) | 0.867 (min 0.745, max 1.069) | 2.364 (min 1.766, max 2.8739) |
| γ-proteobacteria; Pseudohongiellaceae | 1.233E-11 | 4.03 | 0.215 (min 0.006, max 1.138) | 0.188 (min 0, max 1.124) | 0.007 (min 0, max 0.059) | 0.044 (min 0, max 0.620) | 1.776 (min 1.501, max 2.134) | 2.151 (min 1.651, max 2.505) |
| Fusobacteriia; Fusobacteriaceae | 2.760E-08 | 4.03 | 2.134 (min 0.182, max 8.864) | 0.735 (min 0, max 8.335) | 0.113 (min 0, max 0.803) | 0.141 (min 0, max 1.486) | 0.007 (min 0.006, max 0.008) | 0.030 (min 0.022, max 0.038) |
| γ-proteobacteria; Methylophilaceae | 7.296E-10 | 3.98 | 0.154 (min 0.037, max 0.355) | 0.059 (min 0, max 0.211) | 0.155 (min 0.002, max 0.700) | 0.047 (min 0, max 0.667) | 1.591 (min 1.331, max 1.857) | 2.379 (min 2.052, max 2.667) |
| γ-proteobacteria; Pseudoalteromonadaceae | 3.357E-03 | 3.98 | 0.033 (min 0, max 0.133) | 0.020 (min 0, max 0.148) | 0.046 (min 0, max 0.228) | 0.040 (min 0, max 0.276) | 1.997 (min 1.546, max 2.311) | 0.457 (min 0.114, max 0.792) |

**Supplementary Table 5** List of the top 20 significant differentially abundant families observed from oyster and mussel gut and seawater samples obtained in summer and winter as determined using the Kruskal-Wallis rank test (adjusted p-value [FDR] cut-off = 0.01). The Log LDA Score cut-off value was adjusted to 2.0 and significant taxa are given in descending order from the highest to lowest LDA score. Mean abundances (%) are presented for each, with ranges given in parentheses.

| **OTU #** | **Classification** | **FDR** | **LDA score** | **Mean abundance (%)** | | | | | |
| --- | --- | --- | --- | --- | --- | --- | --- | --- | --- |
|  |  |  |  | **Mussels (summer)** | **Mussels (winter)** | **Oysters (summer)** | **Oysters (winter)** | **Seawater (summer)** | **Seawater (winter)** |
| OTU_7 | Mycoplasmataceae; Mycoplasma | 6.218E-13 | 5.34 | 44.247 (min 1.993, max 79.023) | 0.642 (min 0, max 14.171) | 0.434 (min 0, max 2.221) | 0.007 (min 0, max 0.102) | 0.001 (min 0, max 0.003) | 0.008 (min 0.003, max 0.016) |
| OTU_8 | α-proteobacteria; unclassified SAR11 clade Ia | 1.053E-08 | 5.29 | 0.011 (min 0, max 0.041) | 0.183 (min 0.031, max 0.699) | 0.089 (min 0, max 0.649) | 0.850 (min 0, max 15.665) | 36.434 (min 35.379, max 38.311) | 39.404 (min 38.4430, max 40.321) |
| OTU_6 | Mycoplasmataceae; Mycoplasma | 2.459E-15 | 5.08 | 0.008 (min 0, max 0.034) | 0.025 (min 0, max 0.309) | 24.001 (min 0.273, max 64.104) | 0.572 (min 0, max 3.061) | 0.005 (min 0, max 0.010) | 0.006 (min 0, max 0.010) |
| OTU_1 | Halieaceae; Halioglobus | 2.370E-15 | 4.97 | 0.007 (min 0, max 0.038) | 0.036 (min 0, max 0.460) | 15.510 (min 2.041, max 44.221) | 18.806 (min 1.321, max 68.388) | 0.001 (min 0, max 0.004) | 0.003 (min 0, max 0.010) |
| OTU_4 | Mycoplasmataceae; Mycoplasma | 8.564E-16 | 4.9 | 0.017 (min 0, max 0.182) | 0.012 (min 0, max 0.044) | 15.782 (min 0.134, max 39.711) | 2.631 (min 0, max 18.730) | 0.003 (min 0, max 0.006) | 0.001 (min 0, max 0.003) |
| OTU_11 | Mycoplasmataceae; Candidatus Bacilloplasma | 8.564E-16 | 4.87 | 8.335 (min 1.380, max 17.905) | 14.718 (min 0.459, max 42.417) | 0.026 (min 0, max 0.180) | 0.008 (min 0, max 0.088) | - | 0.015 (min 0, max 0.037) |
| OTU_14 | uncultured Mycoplasmataceae | 1.143E-14 | 4.74 | 4.181 (min 0.018, max 19.482) | 11.024 (min 0.177, max 44.534) | 0.016 (min 0, max 0.084) | 0.024 (min 0, max 0.305) | 0.018 (min 0, max 0.055) | 0.004 (min 0, max 0.013) |
| OTU_2 | uncultured Mycoplasmataceae | 1.414E-12 | 4.74 | 0.039 (min 0, max 0.272) | 0.178 (min 0, max 3.366) | 3.860 (min 0.01684, max 43.384) | 10.965 (min 0, max 48.367) | 0.009 (min 0.002, max 0.023) | 0.004 (min 0, max 0.009) |
| OTU_17 | uncultured Mycoplasmataceae | 1.198E-14 | 4.71 | 10.146 (min 1.414, max 36.830) | 0.608 (min 0, max 9.169) | 0.005 (min 0, max 0.098) | 0.001 (min 0, max 0.014) | 0.003 (min 0.002, max 0.008) | 0.023 (min 0, max 0.070) |
| OTU_19 | Mycoplasmataceae; Mycoplasma | 8.869E-16 | 4.56 | 7.198 (min 1.380, max 17.905) | 5.880 (min 0.229, max 18.690) | 0.016 (min 0, max 0.043) | 0.003 (min 0, max 0.024) | 0.002 (min 0.002, max 0.005) | 0.014 (min 0, max 0.043) |
| OTU_13 | Mycoplasmataceae; Mycoplasma | 2.200E-13 | 4.49 | 0.021 (min 0, max 0.117) | 0.004 (min 0, max 0.031) | 6.149 (min 0.021, max 16.718) | 1.876 (min 0, max 10.187) | 0.001 (min 0.002, max 0.003) | - |
| OTU_63 | unclassified Rhodobacteraceae HIMB11 | 3.533E-04 | 4.46 | 0.052 (min 0.003, max 0.255) | 0.061 (min 0.008, max 0.167) | 0.035 (min 0, max 0.103) | 0.082 (min 0, max 0.319) | 5.840 (min 4.436, max 8.359) | 0.305 (min 0.230, max 0.368) |
| OTU_65 | Rhodobacteraceae; Planktomarina | 2.857E-10 | 4.4 | 0.002 (min 0, max 0.010) | 0.069 (min 0, max 0.269) | 0.004 (min 0, max 0.045) | 0.070 (min 0, max 0.882) | 0.007 (min 0.003, max 0.009) | 5.010 (min 2.682, max 7.128) |
| OTU_43 | Rhodobacteraceae; Planktomarina | 9.145E-14 | 4.34 | 0.046 (min 0.008, max 0.116) | 1.583 (min 0.266, max 3.946) | 0.107 (min 0, max 0.037) | 0.074 (min 0, max 0.749) | 0.008 (min 0.004, max 0.013) | 4.379 (min 1.424, max 6.109) |
| OTU_44 | unclassified Flavobacteriaceae NS5 marine group | 1.684E-10 | 4.3 | 0.035 (min 0, max 0.331) | 0.173 (min 0, max 0.674) | 0.008 (min 0.152, max 1.899) | 0.048 (min 0, max 0.900) | 0.838 (min 0.584, max 1.246) | 3.956 (min 3.527, max 4.528) |
| OTU_16 | Pseudomonadaceae; Pseudomonas | 1.838E-12 | 4.28 | 0.043 (min 0.003, max 0.179) | 1.893 (min 0.073, max 25.183) | 0.007 (min 0, max 0.041) | 3.845 (min 0.125, max 20.173) | 0.006 (min 0.003, max 0.010) | 0.006 (min 0.004, max 0.008) |
| OTU_51 | Mycoplasmataceae; Mycoplasma | 7.308E-14 | 4.27 | 3.771 (min 0.151, max 15.604) | 1.403 (min 0, max 14.170) | 0.005 (min 0, max 0.071) | 0.010 (min 0, max 0.104) | 0.013 (min 0, max 0.037) | 0.004 (min 0, max 0.011) |
| OTU_22 | Kiloniellaceae; Kiloniella | 1.353E-11 | 4.27 | 0.001 (min 0, max 0.008) | 0.001 (min 0, max 0.016) | 1.002 (min 0, max 12.784) | 3.721 (min 0, max 31.335) | 0.001 (min 0, max 0.002) | - |
| OTU_1474 | Mycoplasmataceae; Mycoplasma | 1.691E-14 | 4.26 | 0.009 (min 0, max 0.047) | 0.003 (min 0, max 0.017) | 1.782 (min 0.005, max 8.658) | 3.660 (min 0, max 30.868) | - | - |
| OTU_28 | Spirochaetaceae; Spirochaeta 2 | 8.086E-16 | 4.23 | 3.361 (min 0.320, max 14.052) | 0.535 (min 0, max 2.938) | 0.002 (min 0, max 0.033) | 0.002 (min 0, max 0.021) | 0.001 (min 0, max 0.002) | 0.002 (min 0, max 0.005) |

**Supplementary Table 6** List of the top 20 significant differentially abundant OTUs observed from oyster and mussel gut and seawater samples obtained in summer and winter as determined using the Kruskal-Wallis rank test (adjusted p-value [FDR] cut-off = 0.01). The Log LDA Score cut-off value was adjusted to 2.0 and significant taxa are given in descending order from the highest to lowest LDA score. Mean abundances (%) are presented for each, with ranges given in parentheses.
